# Knockdown of RUVBL2 improves hnRNPA2/B1‐stress granules dynamics to inhibit perioperative neurocognitive disorders in aged mild cognitive impairment rats

**DOI:** 10.1111/acel.14418

**Published:** 2024-11-28

**Authors:** Zixuan Wang, Chenyi Yang, Xinyi Wang, Huihui Liao, Xing Liu, Huan Liu, Miao Zhang, Lin Zhang, Haiyun Wang

**Affiliations:** ^1^ The Third Central Clinical College of Tianjin Medical University Tianjin China; ^2^ Department of Anesthesiology The Third Central Hospital of Tianjin Tianjin China; ^3^ Tianjin Key Laboratory of Extracorporeal Life Support for Critical Diseases Tianjin China; ^4^ Artificial Cell Engineering Technology Research Center Tianjin China; ^5^ Nankai University Tianjin China; ^6^ Nankai University Affinity The Third Central Hospital Tianjin China

**Keywords:** aged mild cognitive impairment, dynamics, hnRNPA2/B1, perioperative neurocognitive disorders, RUVBL2, stress granules

## Abstract

Perioperative neurocognitive disorders (PND) is common in aged mild cognitive impairment (MCI) patients and can accelerate the progression to dementia. This process involves heterogeneous nuclear ribonucleoprotein A2/B1 (hnRNPA2/B1)‐mediated aggregates of stress granules (SGs), while RUVBL2 influences the dynamics of these SGs. Our research explored a new target for modulating hnRNAPA2/B1‐SGs dynamics to accelerate their disassembly and potentially delay MCI progression due to PND. We assessed the effect of hippocampal RUVBL2 knockdown on hnRNPA2/B1‐SGs in aged MCI rats through behavioral studies, biochemical experiments and MRI. We also examined hnRNPA2/B1‐SGs dynamics using immunofluorescence staining and fluorescence recovery after photobleaching (FRAP) in rat primary hippocampal neurons. Our results revealed that hnRNPA2/B1 in the hippocampus of aged MCI rats translocates to the cytoplasm to form SGs following anesthesia. RUVBL2 knockdown promotes the disappearance of hnRNPA2/B1‐SGs, allowing hnRNPA2/B1 to return to the nucleus and enhancing functional activity in the brain regions of aged MCI rats. In primary hippocampal neurons, RUVBL2 deletion facilitated hnRNPA2/B1‐SGs transition from hydrogel to liquid, promoting disassembly. We compared three commonly used general anesthetics—3% sevoflurane, 40 mg·kg^−1^·h^−1^ propofol, and 9% desflurane. Sevoflurane upregulated RUVBL2, which decreased the intraneuronal pH and disrupted energy metabolism. These changes resulted in greater stabilization of hnRNPA2/B1‐ SGs. In conclusion, our findings indicated that the knockdown of RUVBL2 expression contributes to the transition of hnRNPA2/B1‐SGs from the hydrogel phase to the liquid phase. Targeted interference with RUVBL2 may represent a novel approach to delay the progression to dementia due to PND in aged MCI patients.

## Graphical abstract

Knockdown of RUVBL2 expression contributes to the transition of hnRNPA2/B1‐SGs from the hydrogel phase to the liquid phase, accelerating their disassembly.

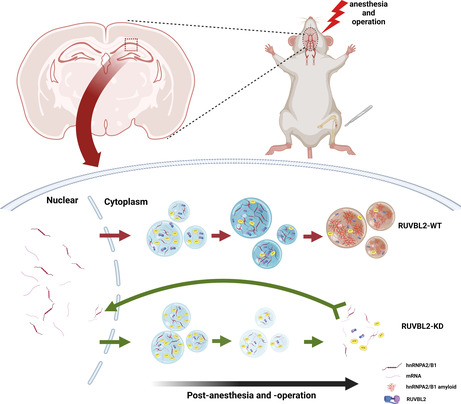

**Abbreviations**
AAV: adeno‐associated virus
ALFF: amplitude of low frequency fluctuation
CCAs: common carotid arteries
CNS: central nervous system
dNCR: delayed neurocognitive recovery
FA: fractional anisotropy
FRAP: fluorescence recovery after photobleaching
hnRNPA2/B1: heterogeneous nuclear ribonucleoprotein A2/B1
IACUC: Institutional Animal Care and Use Committee
Kap‐β2: karyopherin‐β2
LCDs: low‐complexity domains
LLPS: liquid–liquid phase separation
MCI: mild cognitive impairment
MWM: Morris water maze
NOR: novel object recognition
ORIF: open reduction and internal fixation
PND: perioperative neurocognitive disorders
POD: postoperative delirium
PrLD: prion‐like domain
RBPs: RNA‐binding proteins
ROI: region of interest
SD: Sprague–Dawley
SGs: stress granules
siRNA: small interfering RNA

## 1 INTRODUCTION

Perioperative neurocognitive disorders (PND) is one of the most prevalent complications among aged patients undergoing surgery and imposes a significant burden on patients, their families, and the healthcare system (Evered et al., 2018). Mild cognitive impairment (MCI) represents the early stage of decline of various cognitive domains, such as memory, attention, language, executive function, visuospatial skills, and perception (Mian et al., 2024). Aged patients with MCI are more prone to developing PND, which can expedite the progression of MCI to dementia (Fong & Inouye, 2022; Racine et al., 2018). Hence, preventing the hastened progression of MCI to dementia due to PND has the potential to significantly impact the long‐term prognosis of patients but is a formidable challenge.

When subjected to stress, such as heat shock, oxidative stress, viral infection, or radiation, cells rapidly form stress granules (SGs)—membrane‐free organelles containing various proteins and mRNAs—through liquid–liquid phase separation (LLPS) (Hofmann et al., 2021; Hyman & Simons, 2012; Zhang et al., 2024). The formation of SGs is recognized as an adaptive response to transient stress, protecting untranslated mRNAs and certain proteins. However, prolonged stress can lead to the maturation of SGs, potentially leading to the development of more stable pathological forms of SGs that may contribute to neurodegenerative diseases (Wolozin & Ivanov, 2019). Heterogeneous nuclear ribonucleoprotein A2/B1 (hnRNPA2/B1) contains two highly conserved RNA recognition motifs at the N‐terminus and a prion‐like domain (PrLD) at the C‐terminus (Low et al., 2021). The former can increase the affinity of cytoskeleton‐associated protein 5 for RNA during stress and promote LLPS (Falkenberg et al., 2017). The PrLD binds to multiple RNA‐binding proteins (RBPs) through specific sequence recognition, thereby participating in SGs formation (Lee et al., 2016; Lyon et al., 2021; Molliex et al., 2015; Wang et al., 2024). Pathogenic mutations in hnRNPA2B1 have been shown to promote fibril transformation, ultimately leading to the formation of stable cytoplasmic SGs (Kim et al., 2022; Xiang et al., 2015).

Notably, hnRNPA2/B1 is often mutated or abnormally expressed in various neurodegenerative diseases. In disease models, it accumulates abnormally in the cytoplasm of neurons, thereby affecting neuronal function (Davidson et al., 2017; Kim et al., 2013; Kolisnyk et al., 2017). Our previous research confirmed that hnRNPA2/B1 levels increase with increasing severity of central nervous system (CNS) injury in aged rats. Additionally, hnRNPA2/B1 undergoes nucleocytoplasmic transport and accumulates in the cytoplasm, which is associated with a decline in cognitive function (Wang et al., 2020). Subsequently, overexpressing the nuclear transport receptor karyopherin‐β2 (Kap‐β2) in the hippocampus of aged rats with MCI can significantly, although partially, reverse the nucleocytoplasmic mislocalization of hnRNPA2/B1 induced by sevoflurane anesthesia and surgery (Zhang et al., 2023).

RUVBL2, a member of the AAA+ (ATPase associated with diverse cellular activities) superfamily, interacts with its homologue RUVBL1 to increase its stability and form a cyclic heterohexamer with ATPase activity (Parsons & West, 1993). Under stress, the RUVBL1/2 complex binds to nuclear mRNAs and translocates to cytoplasmic SGs, where it sequesters genes regulating glucose metabolism and triggers their inactive translation, leading to impaired cellular energy metabolism (Chen, Hou, et al., 2022). In addition, Jain et al. (2016) performed mass spectrometry and identified RUVBL2 as a core component of SGs that potentially contributes to their stability. However, it remains unknown whether RUVBL2 can influence the progression of PND in MCI rats by modulating hnRNPA2/B1‐SGs dynamics.

In the present study, we aimed to explore the mechanism by which hnRNPA2/B1 plays a role in the development of PND. We observed the formation of hnRNPA2/B1‐SGs in the hippocampus of aged MCI rats subjected to anesthesia and surgery and reported that this effect was correlated with PND‐induced learning and memory impairment. Knockdown of RUVBL2 via stereotactic intracerebral virus injection was observed to delay the fibril formation of hnRNPA2/B1‐SGs induced by anesthesia and surgery and to improve the postoperative learning and memory function of aged MCI rats. In vitro, we verified that RUVBL2 knockdown in rat hippocampal primary neurons resulted in greater mobility and accelerated general anesthetic‐induced disassembly of hnRNPA2/B1‐SGs. In conclusion, our work elucidates the pathogenesis of PND and identifies RUVBL2 as a regulatory target influencing the dynamics of hnRNPA2/B1‐SGs.

## 2 MATERIALS AND METHODS

### 2.1 Animals

All the animals used in this study, that is, 8‐ to 9‐month‐old male Sprague–Dawley (SD) rats (weighing 500–600 g) and gestational day (D)16–18 SD rats (weighing 300–350 g), were obtained from Charles River (Beijing, China). The rats were housed under controlled conditions at a temperature of 24 ± 2°C and humidity of 40%–50% on a 12‐h light/dark cycle. They had free access to water and food and were housed in a comfortable and safe environment. Male rats were raised until they were 22 months old and used to establish the MCI model. These rats then underwent left tibial plateau fracture dissection and internal fixation under general anesthesia. Embryos were taken from D16‐18 SD rats under carbon dioxide anesthesia to extract primary hippocampal neurons. All the rats were monitored twice daily for signs of distress, including significant weight loss (>15%), severe abnormal behavior, or an inability to eat or drink. If these criteria were met, the animals were euthanized using an overdose of anesthetic to prevent unnecessary suffering, in accordance with the humane endpoints approved by the Institutional Animal Care and Use Committee (IACUC). All procedures were approved by the IACUC at the Nankai Animal Resource Center, Nankai University (IACUC number: 2023‐SYDWLL‐000623). The animal husbandry and experimental protocols complied with the principles of laboratory animal care (NIH publication No. 86–23, revised 1985).

### 2.2 Establishment of the aged MCI rat model

The MCI model was established in 22‐month‐old male SD rats (weighing 700–800 g) following the protocol described in our previous study (Wang et al., 2020). Briefly, all the rats were fasted for 12 h before surgery and were anaesthetized via intraperitoneal injection of 0.9% pentobarbital sodium (China National Pharmaceutical Group, Shanghai, China). A 1–2 cm incision was made in the middle of the neck, and the bilateral common carotid arteries (CCAs) were separated from the surrounding sheaths and exposed under a microscope (Nikon, Japan). A syringe needle (diameter of 0.45 mm) was tightly tied to the left and right CCAs at a distance of 1.5 cm from the bifurcation of the CCAs. After careful removal of the needle, the CCAs were returned to their original location. The incision was sutured and sterilized. During the operation, the rectal temperature of the rats was maintained at 36.5–37.5°C using thermostatic blankets. Rats in the sham group were subjected to the same operation to isolate the bilateral CCAs, but the CCAs were not stenosed. The wound was sutured, and 0.3 mg/kg butorphanol (Jiangsu Hengrui Pharmaceuticals Co., Ltd., Jiangsu China) was administered via intraperitoneal injection to provide postoperative analgesia. After surgery, the rats were allowed to recover in a warming box maintained at 28–30°C. They were then housed in a ventilated, air‐conditioned environment and provided adequate food and water. Penicillin (400,000 units) (Hapharm Group Co., Ltd., Heilongjiang China) was intramuscularly injected for 5 days after surgery to prevent infection.

### 2.3 Stereotactic intracerebral injection

The aged MCI rats (weighing 700–800 g), which were fasted for 12 h, were anaesthetized with isoflurane and placed in a stereotaxic frame (RWD, Life Science, Shenzhen, China). A midline incision was made on the scalp, followed by a small craniotomy at the target coordinates selected based on a rat brain atlas. For intrahippocampal injections, two sites were targeted in the same hemisphere. The coordinates were as follows: site 1: −1.7 mm anteroposterior (AP) and ± 1.1 mm mediolateral (ML), and − 2 mm dorsoventral (DV) from the bregma; site 2: −2.9 mm AP and ± 3 mm ML, and − 2.7 mm DV from the bregma. A microliter syringe (Hamilton, Switzerland) equipped with a 33G needle was used for adeno‐associated virus (AAV) injection. The needle was slowly inserted to the target depth (−2 mm or − 2.7 mm DV), and the AAV was injected at a rate of 0.1 μL/min per site. The syringe was preloaded with 1 μL of rAAV‐hSyn‐EGFP‐5′miR‐30a‐shRNA (RUVBL2)‐3′miR‐30a (total virus titer = 5.06 × 10^12^ vg/mL; BrainCase Co., Ltd., Shenzhen, China). The control virus, rAAV‐hSyn‐EGFP‐5′miR‐30a‐shRNA (scramble)‐3′miR‐30a (total virus titer = 5.30 × 10^12^ vg/mL; BrainCase Co., Ltd., Shenzhen, China), was bilaterally injected into the two hippocampal sites in the RUVBL2‐shRNA and Scramble‐shRNA groups. The forward sequence of the shRNA targeting rat RUVBL2 was 5′‐GGA GGA GAC AGA GAT CAT TGA TTC AAG AGA TCA ATG ATC TCT GTC TCC TCC‐3′, and that of the scramble shRNA was 5′‐CCT AAG GTT AAG TCG CCC TCG TTC AAG AGA CGA GGG CGA CTT AAC CTT AGG‐3′. We designed three independent shRNA oligos targeting rat RUVBL2 (Table S1) and selected the one with the highest knockdown efficiency for use throughout the study. After injection, the needle was left in place for an additional 10 min to allow diffusion before being withdrawn. Postoperative recovery, analgesia, infection prevention, and housing conditions for the aged MCI rats were the same as those described in Section 2.2.

### 2.4 Anaesthesia grouping and open reduction and internal fixation (ORIF) surgery

The aged MCI rats were randomly divided into four groups: (1) the control group, which received local anesthesia with 2% lidocaine (China Otsuka Pharmaceutical Co., Ltd); (2) the sevoflurane group, which received 3% sevoflurane (Marushi Pharmaceutical Co., Ltd) via a mask; (3) the propofol group, which received 40 mg·kg^−1^·h^−1^ propofol (AstraZeneca UK Limited) through the tail vein; and (4) the desflurane group, which received 9% desflurane (Baxter Healthcare) via a mask. The total time for surgery and anesthesia was 3 h.

The aged MCI rats (weighing 700–800 g), which were fasted for 12 h, were anaesthetized and fixed on an operating table on a thermostatic blanket. After preparing and disinfecting the skin of the left hind paw, the skin was incised, and the muscle and periosteum were bluntly separated layer by layer to expose the tibia. A transverse fracture of the tibia was induced using surgical forceps, and a 0.3 mm Kirschner wire was inserted into the medullary cavity. In the control group, rats underwent only a skin incision at the tibia under 2% lidocaine local anesthesia, followed by suturing. Postoperative recovery, analgesia, infection prevention, and housing conditions for the aged MCI rats were the same as described in Section 2.2.

### 2.5 Magnetic resonance imaging

On postoperative Days 2 and 30, after sevoflurane anesthesia and ORIF surgery, aged MCI rats in both the RUVBL2‐shRNA group and the Scramble‐shRNA group underwent BOLD‐fMRI and DTI imaging using a Bruker 9.4 T BioSpec 94/30 scanner (Bruker, Ettlingen, Germany). During imaging, each rat was maintained under anesthesia with 1.5%–2% isoflurane, the heart rate and respiratory rate were monitored and the respiratory rate was maintained at 60–70 breaths per minute. Body temperature was maintained at 37.0 ± 0.5°C using a circulating water heating system. A fiber‐optic pulse oximeter sensor attached to the rats' paw was used to monitor blood oxygenation. The rats' heads were immobilized using two ear bars and a bite bar to ensure stability during image acquisition.

First, the instrument was calibrated for wobble and frequency tuning. A refocused echoes (RARE) sequence was subsequently used to confirm the proper positioning of the rat in the scanner. T2‐weighted images of the entire rat brain were acquired as a reference for positioning, with a repetition time (TR) = 2500 ms, echo time (TE) = 33 ms, slice thickness = 1 mm, image size = 256 × 256, and field of view (FOV) = 35 × 35 mm^2^. BOLD‐fMRI data were acquired using an echo planar imaging (EPI) sequence with a TR of 1500 ms, a TE of 20 ms, a slice thickness of 1 mm, an image size of 120 × 80, and an FOV of 24 × 24 mm^2^. DTI data were also collected using an EPI sequence with a TR of 2000 ms, a TE of 22 ms, a slice thickness of 0.8 mm, an image size of 180 × 180, and an FOV of 35 × 35 mm^2^.

### 2.6 Imaging data analysis

fMRI data analysis began with converting the DICOM data into the NIFTI format. After completing the image quality checks and corrections, the first volume of each subject was used as a reference standard. A six‐parameter rigid‐body transformation was applied to spatially realign the remaining volumes, eliminating minor head movements and generating a head‐motion‐corrected mean image. All the subjects' images were aligned voxel‐by‐voxel and normalized to a template space to minimize individual differences. The spatially normalized data were further smoothed with a Gaussian kernel of FWHM [3 3 3] to reduce noise. The data were then low‐pass filtered with a filter range of 0.01–0.1 Hz. Using DPABI software, the amplitude of low frequency fluctuation (ALFF) was calculated for all subjects, with the bilateral hippocampus and bilateral prefrontal cortex serving as seed regions. The ALFF of functional brain regions for all subjects were extracted based on the Paxinos & Watson atlas for rats.

DTI data analysis began with data conversion and image quality checks, followed by adjusting each rat's image origin to the center of the brain. The images were corrected using FSL. Fractional anisotropy (FA) was quantitatively calculated using DKI_TOOLKIT. The rat brain imaging data were spatially normalized. The spatially normalized data were further smoothed with a Gaussian kernel of FWHM [3 3 3] to reduce noise. Quantitative metrics for all subjects' brain regions were extracted based on the Paxinos atlas for rats. Statistical analysis of fMRI and DTI data was performed using two‐sample *t*‐tests.

### 2.7 Morris water maze test

The Morris water maze (MWM) test was performed on both aged MCI rats and sham rats 30 days after bilateral CCAs stenosis to identify aged MCI rats. The test was conducted in a cylindrical tank with a diameter of 170 cm and a height of 50 cm that was filled with opaque water. A platform with a diameter of 12 cm was submerged ~2 cm below the surface in the centre of the third quadrant. Four visual cues were fixed above the edge of the pool. On the first day (D1), the rats were allowed to swim freely for 120 s to acclimatize to the environment. Positioning cruise experiments (spatial acquisition training) were performed for five consecutive days (D2–D6). Each rat underwent four trials per day, during which they were placed in the water at one of the four different quadrants. On the seventh day (D7), the spatial exploration experiment was conducted. The rats that exhibited a 20% decrease in the latency to first cross the platform location and in the ratio of time spent swimming in the target quadrant to total time spent swimming compared with the sham rats were classified as aged MCI rats.

### 2.8 Novel object recognition test

The novel object recognition (NOR) test was performed on control, propofol‐, sevoflurane‐ and desflurane‐treated rats 1 day after ORIF. During the familiarization period (D1), the rats were placed in a 40 × 40 × 40 cm box with a black background and allowed to explore freely for 10 min to familiarize themselves with the environment. During the training period (D2), two identical objects (A1 and A2) were placed adjacent to each other on the floor of the box. The rats were placed in the box with their backs to the two objects. After 10 min, the animals were removed and placed back in their cages. During the test period, one of the identical objects was replaced with a different object (C), and the other was used as the familiar object (A3). We quantified the preference of the rats for the novel object using the discrimination index (DI), which was calculated as (*T*_C−_ *T*_A3_)/(*T*_C_ + *T*_A3_), where *T*_C_ and *T*_A3_ refer to the time spent exploring the novel object and familiar object during the test period, respectively. The objects and the chamber were wiped with 75% alcohol at the end of each experiment to eliminate odor cues.

### 2.9 Barnes maze

The Barnes maze test was performed on control, sevoflurane‐, propofol‐ and desflurane‐treated rats 11 and 30 days after ORIF surgery to assess the postoperative spatial learning and memory abilities of MCI rats. A circular platform with a diameter of 1215 mm surrounded by 18 holes was used. The holes were uniform in diameter (100 mm) and appearance, but only 1 target hole was connected to a black escape box (170 mm long×120 mm wide×60 mm high). On Day 1, the rats were acclimatized for 4 min from the target hole in the escape box. On Day 2, the rats were placed in an opaque cylinder (20 cm diameter×27 cm high) in the center of the maze to restrict their movement for 10 s. A 75.8 dB noise and a white‐bright 1000 liu bulb were delivered as escape stimuli. The rats were trained twice a day for 4 days. On the Day 6, which was the test period, the escape box was removed, and the rats were allowed to freely explore. The escape latency was recorded when the rats first found the hole where the escape box had been and explored it for more than 5 s. From the second training session onwards, the maze was randomly rotated before each training session, but the escape box was always fixed in the same orientation, which prevented the rats from relying on scent to determine the location of the target hole. In addition, the maze and escape box were wiped with 75% alcohol between each session to eliminate residual odors.

### 2.10 Preparation of rat primary hippocampal neurons

Hippocampal neurons were isolated from SD rat embryos at D16‐18. Briefly, pregnant rats were anaesthetized with carbon dioxide, after which the hippocampi of the embryos were dissected and submerged in ice‐cold Hank's solution (Gibco™13150016). The tissues were treated with 0.125% trypsin–EDTA (Gibco, 25200–056) solution at 37°C for 10 min. Neurons were initially plated at a density of 1 × 10^5^ cells/cm^2^ in 6‐ or 24‐well culture plates or glass bottom confocal dishes precoated with 50 μg/mL poly‐D‐lysine (Solarbio, P2100) and cultured in DMEM (Gibco, C11559900) supplemented with 10% fetal bovine serum (Gibco, A5669701) and 50 U/mL penicillin–streptomycin (Gibco, 15070063). The culture dishes were placed in a humidified incubator containing 5% CO_2_ and 95% air at 37°C, and after 4 h, the culture medium was completely replaced with neurobasal medium (Gibco, 21103049) containing 1% L‐glutamine (Gibco, 35050–061) and 2% B27 (Gibco, 17504–044). After three days, half of the neuronal medium was replaced with medium containing 10 μmol/L cytarabine to inhibit the growth of glial cells. Thereafter, half of the medium was replaced with fresh neuronal medium every 3 days.

### 2.11 Small interfering RNA (siRNA) construction and transfection

RUVBL2‐siRNA, which was transfected into neurons to downregulate the expression of the RUVBL2 gene (NM_001025405.3), was designed by GenePharma Co., Ltd. (Shanghai, China). The sense and antisense sequences of the siRNA targeting rat RUVBL2 were 5′‐GCC CGA GAC UAU GAU GCC ATT‐3′ and 5′‐UGG CAU CAU AGU CUC GGG CTT‐3′, respectively, and those of the negative control (NC) siRNA were 5′‐UUC UCC GAA CGU GUC ACG UTT‐3′ and 5′‐ACG UGA CAC GUU CGG AGA ATT‐3′. NC‐siRNA did not target RUVBL2. Neurons were transfected after 21 days of culture in vitro, when they reached senescence. For primary hippocampal neuron transfection, 50 nM siRNA was added to Opti‐MEM I Reduced Serum Medium (Gibco, 31985070). RUVBL2‐siRNA or an equal amount of NC‐siRNA mixed with Lipofectamine® 2000 Reagent (Invitrogen, 11668019) was added to the primary hippocampal neurons. The neurons were then incubated at 37°C with 5% CO_2_. Six hours after transfection, the medium was replaced with Neurobasal medium containing glutamine and B27. After 48 h, the transfected neurons were analyzed by Western blotting to confirm RUVBL2 knockdown. We designed three siRNA sequences targeting rat RUVBL2 (Table S2) and selected the one with the highest knockdown efficiency for use throughout the study.

### 2.12 Establishment of the chronic hypoxia model and grouping

Chronic hypoxia models were generated after siRNA transfection. The culture dishes were transferred to an incubator containing 3% O_2_, 92% N_2_ and 5% CO_2_ for 3 h. Then, the hypoxia‐exposed neurons were treated with 3% sevoflurane for 3 h or left untreated (as the hypoxia control group). Sevoflurane was delivered into the incubator at a flow rate of 2 L/min. The concentration of sevoflurane in the chamber was monitored using an anesthesia analyzer (Datex‐Ohmeda, UK). When the concentration of sevoflurane reached 3%, the neurons were placed in the incubator for 3 h.

Neurons were divided into four groups to explore the impact of RUVBL2 depletion on the dynamics of hnRNPA2/B1‐SGs formed in response to sevoflurane exposure: (1) the hypoxia‐exposed and NC‐siRNA‐transfected (NC‐siRNA‐Ctrl) group; (2) the hypoxia‐exposed, NC‐siRNA‐transfected and 3% sevoflurane‐exposed (NC‐siRNA‐Sevo) group; (3) the hypoxia‐exposed and RUVBL2‐siRNA‐transfected (RUVBL2‐siRNA‐Ctrl) group; and (4) the hypoxia‐exposed, RUVBL2‐siRNA‐transfected and 3% sevoflurane‐exposed (RUVBL2‐siRNA‐Sevo) group.

### 2.13 Fluorescence recovery after photobleaching (FRAP)

The hnRNPA2B1‐mCherry plasmid (GenePharma, Co., Ltd.) and RUVBL2‐siRNA were co‐transfected into primary neurons with Lipofectamine® 2000 Reagent according to the manufacturer's instructions on day in vitro 21. At 48 h after transfection, the neurons were exposed to chronic hypoxia and 3% sevoflurane for 3 h to induce hnRNPA2/B1‐SG formation. FRAP was used to assess the mobility of hnRNPA2B1‐SGs via LLPS via a laser scanning confocal microscope (Olympus FV1000) and a 100× oil immersion objective. The region of interest (ROI) was photobleached using a 594 nm FRAP laser at 100% power to excite mCherry‐hnRNPA2/B1, and time‐lapse imaging was performed using a 594 nm imaging laser. After baseline images were acquired, the ROI was bleached at 100% power for 10 s. The recovery time was 200 s, and images were acquired every 24 s.

### 2.14 Immunofluorescence

Rat brain tissue fixed with paraformaldehyde was subjected to gradient dehydration with 10%, 20% and 30% sucrose solutions. The tissue was subsequently embedded in OCT and sectioned at a thickness of 20 μm using a freezing microtome. After chronic hypoxia exposure, primary hippocampal neurons exposed to sevoflurane were fixed with ice‐cold 4% paraformaldehyde for 15 min at room temperature. The samples were permeabilized with 0.3% Triton X‐100 (Merck, T8787) in PBS for 20 min, blocked with 5% Normal Goat Serum (Invitrogen, R37624) for 1 h, and then incubated overnight at 4°C with the following primary antibodies: anti‐hnRNPA2/B1 (Abcam, ab259894), anti‐TIA1 (Santa Cruz Biotechnology, Sc‐166247), anti‐RUVBL2 (ORIGENE, TA504281), and anti‐MAP2 (Abcam, Ab5392). After being rewarmed at room temperature for 1 h, the samples were washed with PBS‐Tween 20 three times for 5 min each. The sections or cells were labeled with the following fluorescent dye‐conjugated secondary antibodies for 1 h at 37°C in PBS‐Tween 20 containing 1% goat serum: Alexa Fluor® 647‐conjugated goat anti‐chicken (Abcam, ab150171), Alexa Fluor® 555‐conjugated goat anti‐rabbit (Abcam, ab150078), Alexa Fluor® 488‐conjugated goat anti‐mouse (Abcam, ab150113), Alexa Fluor® 594‐conjugated goat anti‐mouse (Abcam, ab150116), Alexa Fluor® 488‐conjugated goat anti‐rabbit (Abcam, ab150077) and Congo Red (Biotium, 80,028). The samples were subsequently washed with PBS‐Tween 20. Nuclei were stained using 4,6‐diamidino‐2‐phenylindole (DAPI) (Invitrogen, D3571) for 8 min at room temperature, and the sections and cells were sealed with anti‐fluorescent quenching sealing agent. Images were captured with a Zeiss LSM 900 confocal laser scanning microscope (with a 63× objective). The number and diameter of SGs in primary hippocampal neurons were determined using ImageJ. Two independent researchers, who were blinded to the experimental groups, manually quantified the number and diameter of cytoplasmic SGs. Only SGs located within the cytoplasm were included in the analysis. If significant discrepancies were observed between the two sets of counts, a third blinded researcher performed an additional round of counting to resolve any inconsistencies and ensure accuracy.

### 2.15 Co‐immunoprecipitation

For Co‐immunoprecipitation, we used an Immunoprecipitation Kit with Protein A+G Agarose Gel (Beyotime, P2197M). Briefly, hypoxia‐exposed rat primary hippocampal neurons were treated with or without sevoflurane for 3 h. Subsequently, the cells were washed three times with PBS and lysed on ice in lysis buffer containing protease inhibitors and phosphatase inhibitors. The supernatant was collected by centrifuging the cell lysate at 4°C and 12,000 × *g* for 5 min. Agarose beads preconjugated with an anti‐hnRNPA2/B1 antibody for IP (Abcam, ab183654) or a rabbit IgG isotype control antibody (Arigo, ARG65346) were incubated with the supernatant with rotation for 1 h. The agarose beads were washed three times with TBS, the supernatant was discarded, and the agarose beads were resuspended. The samples were incubated at 4°C overnight with Protein A+G Agarose Beads conjugated to an IP antibody or IgG. The agarose beads were centrifuged, and the resulting complexes were eluted from the beads using lysis buffer containing protease and phosphatase inhibitors. The samples were subsequently boiled in SDS–PAGE Sample Loading Buffer and analyzed by Western blotting. The immunoprecipitated proteins were separated by SDS–PAGE, and target proteins or interacting proteins were detected with specific antibodies.

### 2.16 Western blotting

RIPA lysis buffer containing protease inhibitors was added to rats hippocampal tissue or primary hippocampal neurons from each group to extract proteins. Nuclear and cytoplasmic proteins were extracted with a protein extraction kit (Thermo Scientific™, 78835) according to the manufacturer's protocol. After quantification of the protein concentration by a BCA Assay Kit (Beyotime, P0012), 15 μg of each protein sample was separated by SDS–PAGE and transferred to PVDF membranes. The PVDF membranes were blocked with 5% skim milk in TBS‐Tween 20 and then incubated overnight at 4°C with the following primary antibodies: anti‐RUVBL2 (Abcam, ab196027), anti‐hnRNPA2/B1 (Abcam, ab259894), anti‐HIF1α (Abmart T55824), anti‐β‐Actin (Abcam, Ab8226), anti‐β‐Tubulin (Affinity, T0023), anti‐GAPDH (Abcam, ab128915), and anti‐HDAC1 (Abcam, ab109411). The membranes were washed with TBS‐Tween 20 and incubated with horseradish peroxidase‐labelled secondary antibody (Affinity, S0001) for 1 h at room temperature. After thorough washing of the membranes again, the chemiluminescent signals were detected using a Super Excellent Chemiluminescent Substrate (ECL) Detection Kit (Elabscience, E‐IR‐R308), and the blots were imaged using an enhanced chemiluminescence system. ImageJ software was used to analyze the expression level of the target proteins by calculating the ratio of the grayscale value of the target protein band to that of the internal reference band.

### 2.17 Quantitative real‐time PCR

Total RNA was extracted from the hippocampus of postoperative aged MCI rats using the Trizol method. The concentration and purity of the extracted RNA were determined using a NanoDrop spectrophotometer (NanoDrop Technologies). Subsequently, RNA samples were reverse transcribed into cDNA utilizing the PrimeScriptTM RT Master Mix kit (Takara, RR036Q). For the amplification process, reaction solutions were prepared with the TB GreenTMPremix Ex Taq™ II Kit (Takara, RR820A) and amplified through a two‐step PCR protocol using the QuantStudio™ 5 Flex Real‐Time PCR System (ThermoFisher, USA). β‐actin served as the internal control. Relative gene expression levels were calculated using the 2−^ΔΔCt^ method. The primers used in the study were as follows: RUVBL2: Forward primer: 5′‐TGAGCTCAAAGGTGAAACAATGG‐3′, Reverse primer: 5′‐AGAAAACAGGCTGGGAAGGG‐3′; β‐Actin: Forward primer: 5′‐GCTGTGCTATGTTGCCCTAGACTTC‐3′, Reverse primer: 5′‐GGAACCGCTCATTGCCGATAGTG‐3′.

### 2.18 Intracellular pH measurement

The intracellular pH was measured utilizing 2′,7′‐bis‐(2‐carboxyethyl)‐5‐(and‐6)‐carboxyfluorescein and acetoxymethyl ester (BCECF AM) (Beyotime, S1006), a fluorescent probe capable of permeating cell membranes. Neurons were incubated with BCECF‐AM in serum‐free DMEM at a ratio of 1:1000 for 20 min in a 37°C incubator according to the manufacturer's instructions. The neurons were subsequently washed three times with serum‐free DMEM. For the pH assay, the fluorescence intensity was measured using a fluorescence microplate reader (Thermo Scientific, Waltham, MA, USA) at an excitation wavelength of 488 nm and an emission wavelength of 535 nm.

### 2.19 ATP level measurement

The ATP content in hippocampal tissue was quantified using an Enhanced ATP Assay Kit (Beyotime, China, S0027) in accordance with the manufacturer's instructions. Briefly, the assay buffer was gently mixed with the substrate at room temperature. The mixture (100 μL) was added to each well and then incubated with shaking for 15 min at room temperature. The tissue homogenate supernatant and 100 μL ATP assay working solution were added to a 96‐well clear‐bottom black plate and mixed thoroughly, and then, the luminescence was measured with a microplate luminometer (Thermo Scientific, Waltham, MA, USA). Subsequently, the protein concentration of the supernatant was quantified using a BCA Protein Assay Kit (Beyotime, China, P0012). The ATP concentration is expressed as nmol/mg protein.

ATP levels in primary hippocampal neurons were measured using pCMV‐AT1.03 (ATP‐specific fluorescent probe) (Beyotime, D2604). Primary hippocampal neurons were first plated at approximately 0.3 × 105 cells/well in 96‐well clear‐bottom black polystyrene microplates. The neurons were co‐transfected with siRNA and the ATP‐specific fluorescent probe and exposed to drugs, and the fluorescence intensity was measured using a fluorescence microplate reader (Thermo Scientific, Waltham, MA, USA) at an excitation wavelength of 435 nm and an emission wavelength of 527 nm. The value of the control group was used as the baseline, and the ratios of the ATP level in the sevoflurane group to that in the control group was calculated to compare the effects of different drugs on ATP levels.

### 2.20 Statistical analyses

*t* test was used for comparisons between two groups. One‐way analysis of variance (ANOVA) was used for comparisons among multiple groups. FRAP results were analyzed using two‐way ANOVA to assess the difference in fluorescence intensity between groups at each time point. For colocalization analysis, Pearson's correlation coefficient was used. All statistical analyses were performed using GraphPad Prism 9.5.1 software. A *P* value less than 0.05 was considered to indicate statistical significance (*p < 0.05, **p < 0.01, ***p < 0.001 and ****p < 0.0001).

## 3 RESULTS

### 3.1 hnRNPA2/B1 localizes to SGs in aged MCI rats after surgery with diverse general anaesthetics

hnRNPA2B1 is known to transport many pre‐mRNAs from the nucleus to the cytoplasm under stress (Ravanidis et al., 2018). We first constructed aged MCI rats using previously established methods to determine whether hnRNPA2B1 mediates PND in aged MCI rats by participating in the recruitment of SGs (Wang et al., 2020). In the MWM test, both the latency to escape and the ratio of the time spent swimming in the target quadrant to the total swimming time increased by less than 20% in aged MCI rats compared with those in sham‐operated rats (Figure S1a–c).

**FIGURE 1:**
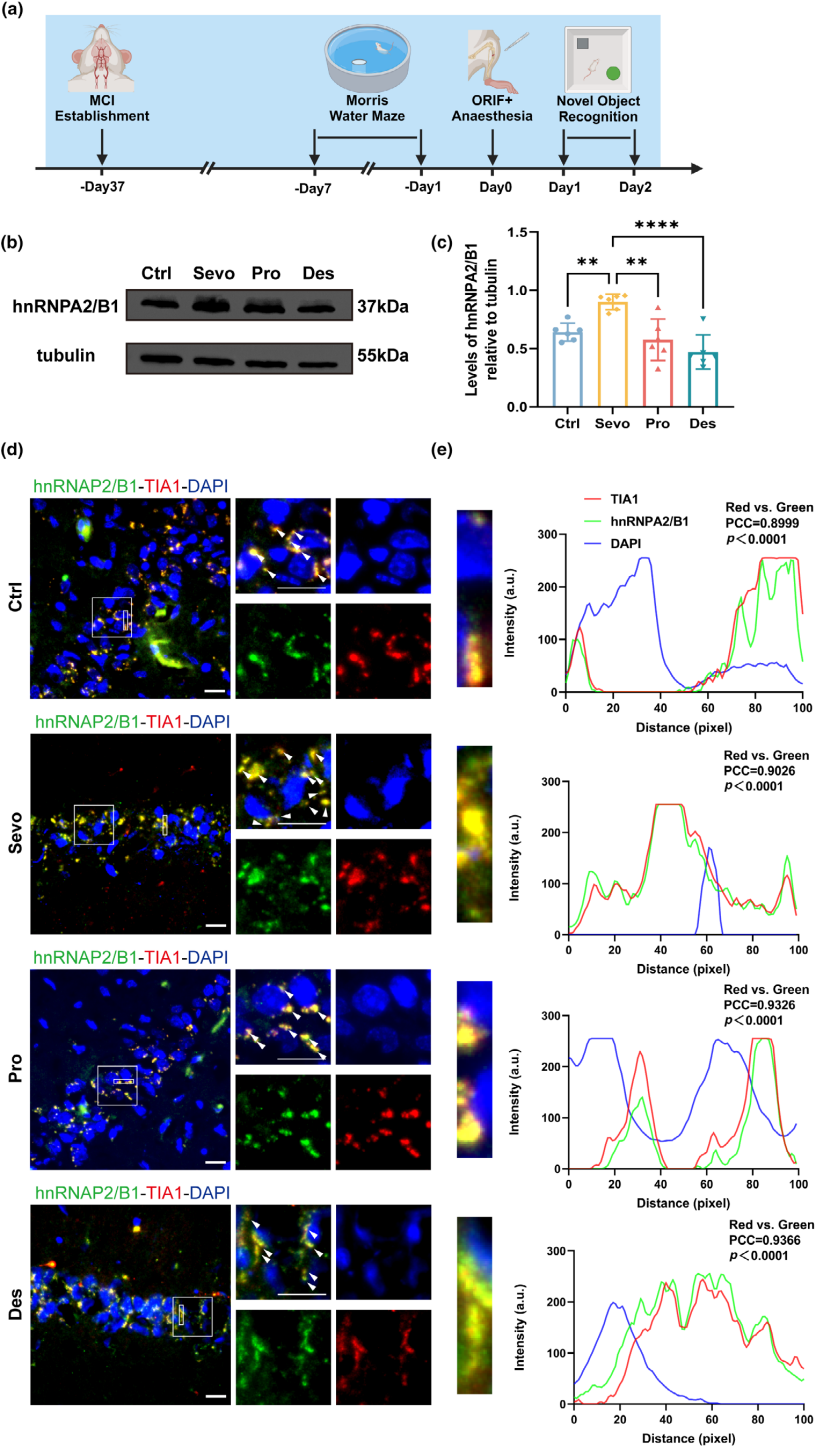
hnRNPA2/B1 localizes to SGs in MCI rats after surgery with diverse general anesthetics. (a) Timeline of the experiment. (b, c) Representative Western blot and statistical histogram of hippocampal lysates from sevoflurane‐, propofol‐, and desflurane‐anaesthetized MCI rats and postoperative aged MCI rats showing hnRNPA2/B1 expression. Western blots were labelled with hnRNPA2/B1 and β‐tubulin antibodies (*n* = 6 independent experiments, one‐way ANOVA followed by the post hoc Bonferroni multiple comparisons test). (d) Representative confocal microscopy images depicting the expression of hnRNPA2/B1 and TIA1 in the hippocampal CA1 region of aged MCI rats after sevoflurane, propofol and desflurane anesthesia and surgery. (e) Fluorescence co‐localization analysis of hnRNPA2/B1, TIA1 and DAPI within the rectangular region of interest in Figure 1c (PCC: Pearson's correlation coefficient). **p < 0.01, ****p < 0.0001. Values are presented as the means ± SEMs. Scale bar = 20 μm.

Aged MCI rats were subjected to ORIF under 3% sevoflurane, 40 mg·kg^−1^·h^−1^ propofol, or 9% desflurane anesthesia. These three general anesthetics are commonly used in clinical anesthesia, and their concentration were selected based on their ability to tolerate surgical stimuli and equivalent dosage conversions (Logginidou et al., 2003; Miller, 2015). Western blot demonstrated that hnRNPA2/B1 expression was highest in the hippocampus of aged MCI rats in the sevoflurane group (Figure 1b,c). According to our previous study, the more severe the CNS injury is, the higher the level of hnRNPA2/B1 expression (Wang et al., 2020), suggesting that sevoflurane is more neurotoxic than other general anesthetics are. This finding was also corroborated by the NOR test results, which indicated that rats in the sevoflurane group spent less time exploring the novel object (Figure S1d).

Our previous study showed that hnRNPA2/B1 can translocate from the nucleus to the cytoplasm and aggregate in the hippocampus of aged MCI rats after anesthesia and ORIF (Zhang et al., 2023). We performed co‐staining for hnRNPA2/B1 and the SGs core protein TIA1 to determine whether hnRNPA2/B1 is involved in anesthesia‐ and surgery‐induced recruitment of SGs and found that hnRNPA2/B1 robustly colocalized with TIA1 in the control, sevoflurane, propofol, and desflurane anesthesia groups (Figure 1d–e). Therefore, hnRNPA2/B1 is a significant constituent of SGs formed in response to anesthesia and surgery.

### 3.2 RUVBL2 knockdown promotes the translocation of hnRNPA2/B1 to the nucleus and the dissipation of hnRNPA2/B1‐SGs

**FIGURE 2:**
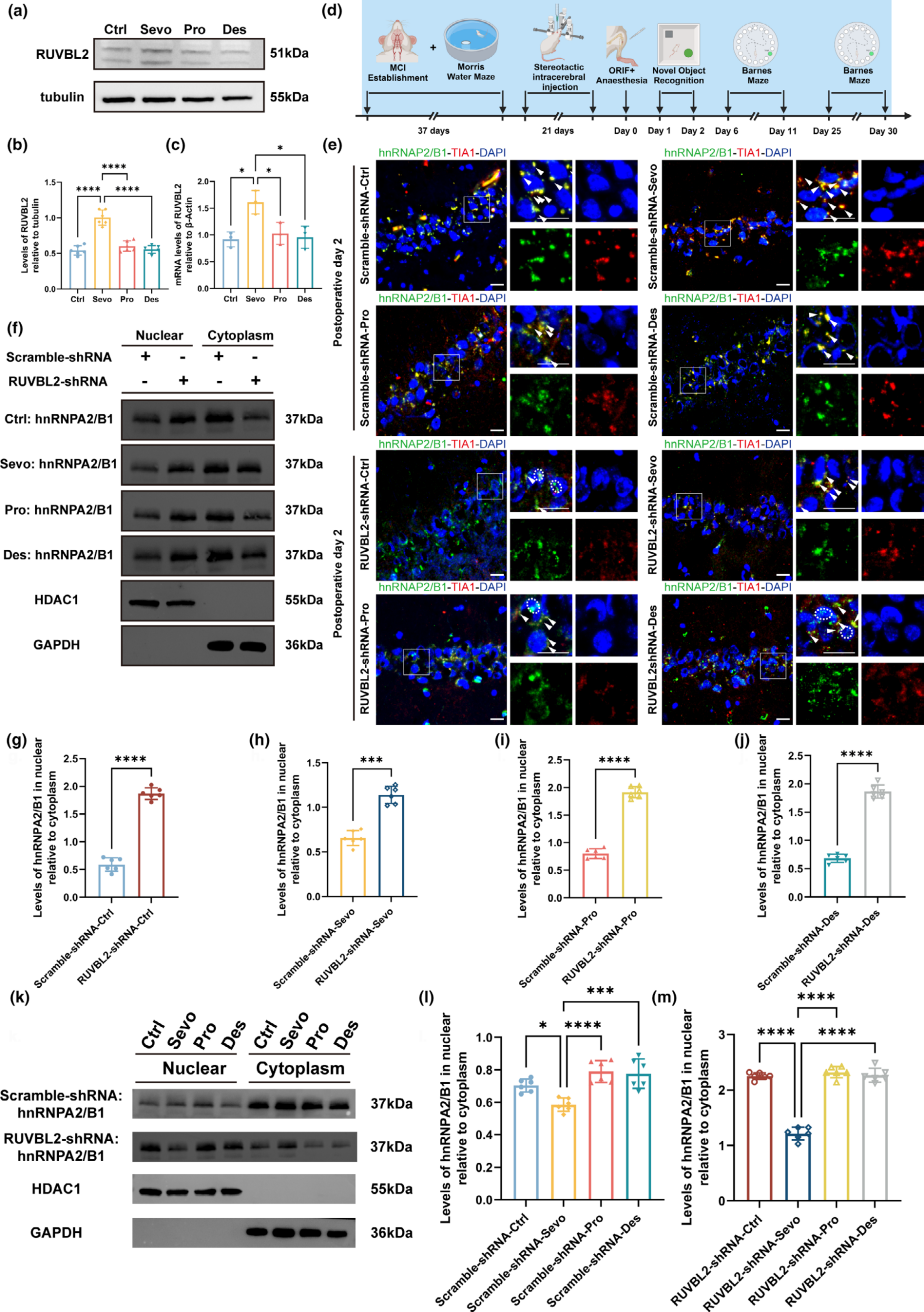
RUVBL2 knockdown promotes the return of hnRNPA2/B1 to the nuclei and dissipation of hnRNPA2/B1‐SGs. (a, b) Representative Western blot and statistical histogram of hippocampal lysates from sevoflurane‐, propofol‐, and desflurane‐anesthetized MCI rats and postoperative aged MCI rats showing RUVBL2 expression. Western blots were labelled with RUVBL2 and β‐tubulin antibodies (*n* = 6 independent experiments, one‐way ANOVA followed by the post hoc Bonferroni multiple comparisons test). (c) RUVBL2 mRNA levels after sevoflurane, propofol, and desflurane anesthesia and surgery (*n* = 3 independent experiments, one‐way ANOVA followed by the post hoc Bonferroni multiple comparisons test). (d) Timeline of the experiment. (e) Representative confocal microscopy images of hnRNPA2/B1 and TIA1 expression in the hippocampal CA1 region of aged MCI rats in the Scramble‐shRNA group on Day 2 after anesthesia and surgery. (f) Representative Western blot of hippocampal nuclei and cytoplasmic lysates showing hnRNPA2/B1 expression in aged MCI rats on Day 2 after sevoflurane, propofol, or desflurane‐anesthetized and surgery. Western blots were labelled with hnRNPA2/B1, HDAC1 and GAPDH antibodies. HDAC1 was used as a nuclei internal reference protein, and GAPDH was used as a cytoplasmic internal reference protein. (g–j) hnRNPA2/B1 nucleoplasmic ratio on Day 2 after the post‐anesthesia and surgery Day 2 (*n* = 6 independent experiments, unpaired *t* test). (k–m) Representative Western blot and statistical histogram of hippocampal nuclei and cytoplasmic lysates showing hnRNPA2/B1 expression in aged MCI rats on Day 2 after sevoflurane, propofol, or desflurane‐anesthetized and surgery. Western blots were labelled with hnRNPA2/B1, HDAC1, and GAPDH antibodies. HDAC1 was used as a nuclei internal reference protein, and GAPDH was used as a cytoplasmic internal reference protein. (*n* = 6 independent experiments; one‐way ANOVA followed by the post hoc Bonferroni multiple comparisons test). *p < 0.05, ***p < 0.001 and ****p < 0.0001; values are presented as the means ± SEMs.

RUVBL2 and its homologue RUVBL1 act as ATPases when assembled into the heterohexamer RUBVL1/2 complex, which is extensively involved in cellular physiological processes, but is inactive when expressed individually (Nano & Houry, 2013). Given the involvement of RUVBL2 in regulating NaAsO2‐induced SGs formation in mammalian cells (Jain et al., 2016), we investigated the gene and protein levels of RUVBL2 in the hippocampus of aged MCI rats that underwent ORIF. The PCR and Western blot results demonstrated increased RUVBL2 mRNA and protein expression in the hippocampus of aged MCI rats subjected to ORIF under sevoflurane anesthesia (Figure 2a–c).

We next wanted to determine whether RUVBL2 knockdown affects the formation of hnRNPA2/B1‐SGs in the hippocamps after anesthesia and surgery in aged MCI rats. We achieved this objective by first administering an AAV carrying an shRNA targeting RUVBL2 into the hippocampus of aged MCI rats via stereotaxic intracerebral injection. We tested three independent RUVBL2 shRNA oligos, all of which effectively downregulated RUVBL2 expression in the hippocampus of aged MCI rats compared to Scramble‐shRNA. Among them, RUVBL2‐shRNA‐#2 showed the highest efficiency, and was therefore selected for further studies (Figure S1e,f). Then, the aged MCI rats were subjected to general anesthesia and ORIF. The results demonstrated that on the second postoperative day, hnRNPA2/B1 in the hippocampus of rats in the Scramble‐shRNA group translocated to the nucleus and formed hnRNPA2/B1‐SGs in the perinuclear area (Figure 2e). In contrast, while hnRNPA2/B1‐SGs were also detected in the perinuclear area of the hippocampus in rats in the RUVBL2‐shRNA group, some hnRNPA2/B1 had returned to the nucleus (Figure 2e). Consistent with this finding, the hnRNPA2/B1 nucleoplasmic ratio was greater in the RUVBL2‐shRNA group than in the Scramble‐shRNA group (Figure 2f–j). Furthermore, the hnRNPA2/B1 nucleoplasmic ratio was consistently lower in sevoflurane‐treated rats than in control‐, propofol‐, and desflurane‐treated rats in both the scramble‐shRNA and RUVBL2‐shRNA groups (Figure 2k,m). These findings indicates that sevoflurane is more neurotoxic than the other anesthetics are.

**FIGURE 3:**
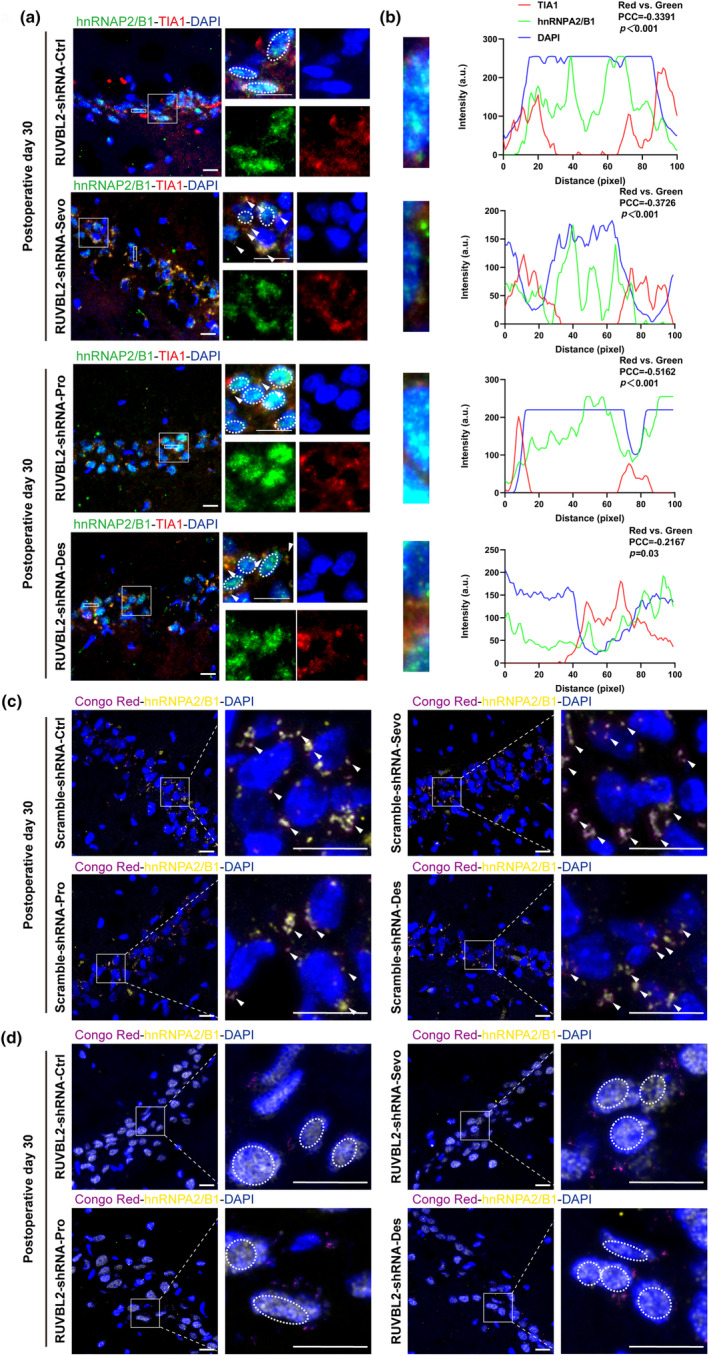
RUVBL2 knockdown reduces hnRNPA2/B1 and TIA1 co‐localization, promotes hnRNPA2/B1 nucleation, and improves hnRNPA2/B1 fibril formation (a) Representative confocal microscopy images of hnRNPA2/B1 and TIA1 expression in the hippocampal CA1 region of the RUVBL2‐shRNA group of aged MCI rats on Day 30 after anesthesia and surgery. (b) Fluorescence co‐localization analysis of hnRNPA2/B1, TIA1 and DAPI within the rectangular region of interest in (a) (PCC: Pearson's correlation coefficient). Scale bar = 20 μm. (c) Representative confocal microscopy images of Congo Red staining and hnRNPA2/B1 expression in the hippocampal CA1 region of the Scramble‐shRNA group of aged MCI rats on Day 30 after anesthesia and surgery. (d) Representative confocal microscopy images of Congo Red staining and hnRNPA2/B1 expression in the hippocampal CA1 region of the RUVBL2‐shRNA group of aged MCI rats on Day 30 after anesthesia and surgery. Scale bar = 20 μm.

PND encompasses both postoperative delirium (POD) within 1 week after surgery, characterized by fluctuating levels of consciousness and impaired attention, and delayed neurocognitive recovery (dNCR) within 30 days after surgery, characterized by impaired memory, learning, and attention. Thus, we studied the hippocampus of aged MCI rats on postoperative Day 11 and postoperative Day 30. We found that in the Scramble‐shRNA group, hnRNPA2/B1 formed hnRNPA2/B1‐SGs in the perinuclear region on postoperative Days 11 and 30, as well as on postoperative Day 2 (Figure S2a,b). In contrast, in the RUVBL2‐shRNA group, hnRNPA2/B1 gradually returned to the nucleus over time and was present in the nucleus on postoperative Day 11 (Figure S2c); concomitantly, the colocalization of hnRNPA2/B1 with TIA1 in the perinuclear area was decreased on postoperative Day 30 (Figure 3a,b). The observed nucleoplasmic distribution of hnRNPA2/B1 on postoperative day 11 (Figure S3a,c–f, k,m) and postoperative Day 30 (Figure S3b, g–j, n–p) was consistent with the above findings.

### 3.3 RUVBL2 knockdown reduces hnRNPA2/B1‐mediated fibril formation

Prolonged stress and anomalous disassembly of SGs amplify interactions among their contents, prompting SGs to transition from the liquid phase to hydrogel phase or even solid phase (fibril formation). We characterized fibril formation using Congo red and hnRNPA2/B1 staining. On postoperative Day 30, perinuclear hnRNPA2/B1 aggregates in the Scramble‐shRNA group became fibrotic, whereas hnRNPA2/B1 returned to the nucleus and did not become fibrotic in the RUVBL2‐shRNA group (Figure 3c,d).

### 3.4 RUVBL2 knockdown increases ATP levels and improves learning and memory in aged MCI rats after anaesthesia and surgery

**FIGURE 4:**
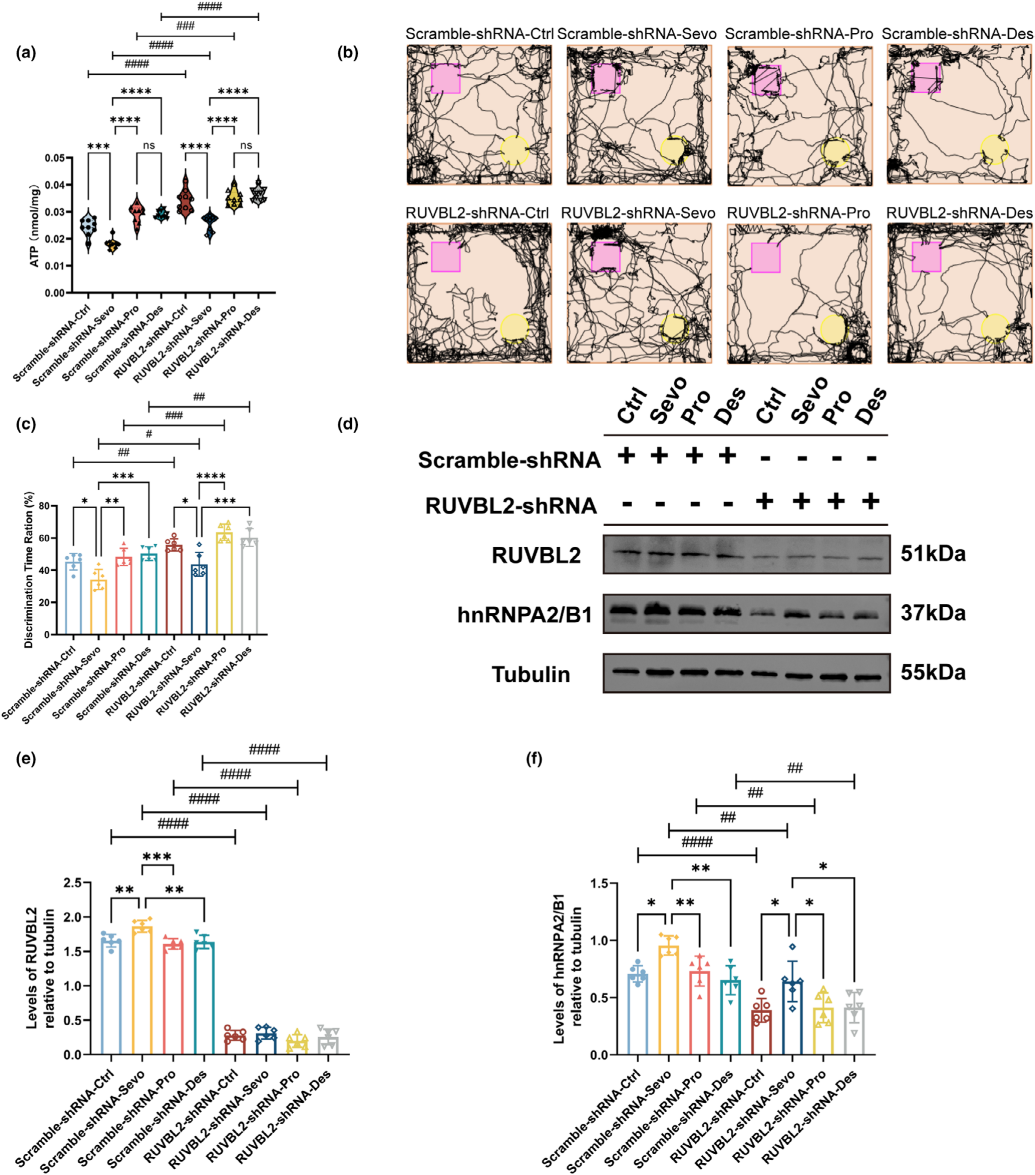
RUVBL2 knockdown increases ATP levels and improves learning and memory in MCI rats after anesthesia and surgery. (a) ATP levels in the hippocampus of aged MCI rats on Day 2 after post‐anesthesia and surgery (*n* = 9 independent experiments, unpaired *t* test and one‐way ANOVA followed by the post hoc Bonferroni multiple comparisons test). (b) NOR test trajectory map of aged MCI rats on Day 2 after anesthesia and surgery. (c) Ratio of the discrimination time for novel objects in the NOR test on Day 2 after anesthesia and surgery (*n* = 6 independent experiments, unpaired *t* test and one‐way ANOVA followed by the post hoc Bonferroni multiple comparisons test). (d–f) Representative Western blots and statistical histogram of hippocampal lysates showing the expression of hnRNPA2/B1 and RUVBL2 from aged MCI rats expressing hnRNPA2/B1 on Day 2 after anesthesia and surgery. Western blots were labelled with hnRNPA2/B1, RUVBL2 and β‐tubulin antibodies (*n* = 6 independent experiments, unpaired *t* test and one‐way ANOVA followed by the post hoc Bonferroni multiple comparisons test). *p < 0.05, **p < 0.01, ***p < 0.001, ****p < 0.0001, #*p* < 0.05, ##*p* < 0.01, ###*p* < 0.001 and ####*p* < 0.0001; values are presented as the means ± SEMs.

During cellular stress, the RUVBL1/2 complex shows increased activity and is concentrated in SGs, inhibiting the translation of mRNAs that regulate energy metabolism within SGs. Consequently, cellular energy metabolism is impaired (Chen, Qin, et al., 2022). We found that ATP levels in the hippocampus of rats in the RUVBL2‐shRNA group were elevated on postoperative day 2, suggesting an improvement in energy metabolism (Figure 4a); moreover, ATP levels were lower in the sevoflurane group than in the other groups (Figure 4a). However, on postoperative Days 11 (Figure S4) and 30 (Figure S4b), we observed similar ATP levels in the RUVBL2‐shRNA and Scramble‐shRNA groups.

We assessed the memory of rats by the NOR test on postoperative Day 2. First, rats in the RUVBL2‐shRNA group explored the novel object for a longer amount of time than did those in the Scramble‐shRNA group, irrespective of whether they were treated with the control sevoflurane, propofol, or desflurane (Figure 4b,c). Furthermore, rats in the sevoflurane group exhibited shorter exploration times for the novel object than did those in the control, propofol, and desflurane groups, regardless of whether RUVBL2 was knocked down (Figure 4c). The results of Western blot analysis of hnRNPA2/B1 expression in the rat hippocampus on postoperative Day 2 were consistent with the NOR test results (Figure 4d). hnRNPA2/B1 expression was highest in rats receiving sevoflurane anesthesia regardless of whether RUVBL2 was knocked down or not, and hnRNPA2/B1 expression was lower in the RUVBL2‐shRNA group than in the Scramble‐shRNA group (Figure 4f). And, we found that in the Scramble‐shRNA group, RUVBL2 was elevated in rats receiving sevoflurane anesthesia (Figure 4e). The Barnes maze test was conducted on postoperative Days 11 and 30, and the results were comparable to those observed on postoperative Day 2. Compared with rats in the Scramble‐shRNA group, rats in the RUVBL2‐shRNA group exhibited enhanced spatial learning and memory functions (Figure S4c,d). The results of Western blot analysis of hnRNPA2/B1 expression in the hippocampus on postoperative Days 11 (Figure S4e,g) and 30 (Figure S4h–j) were also validated.

### 3.5 MRI revealed that RUVBL2 knockdown increases ALFF and FA in aged MCI rats

**FIGURE 5:**
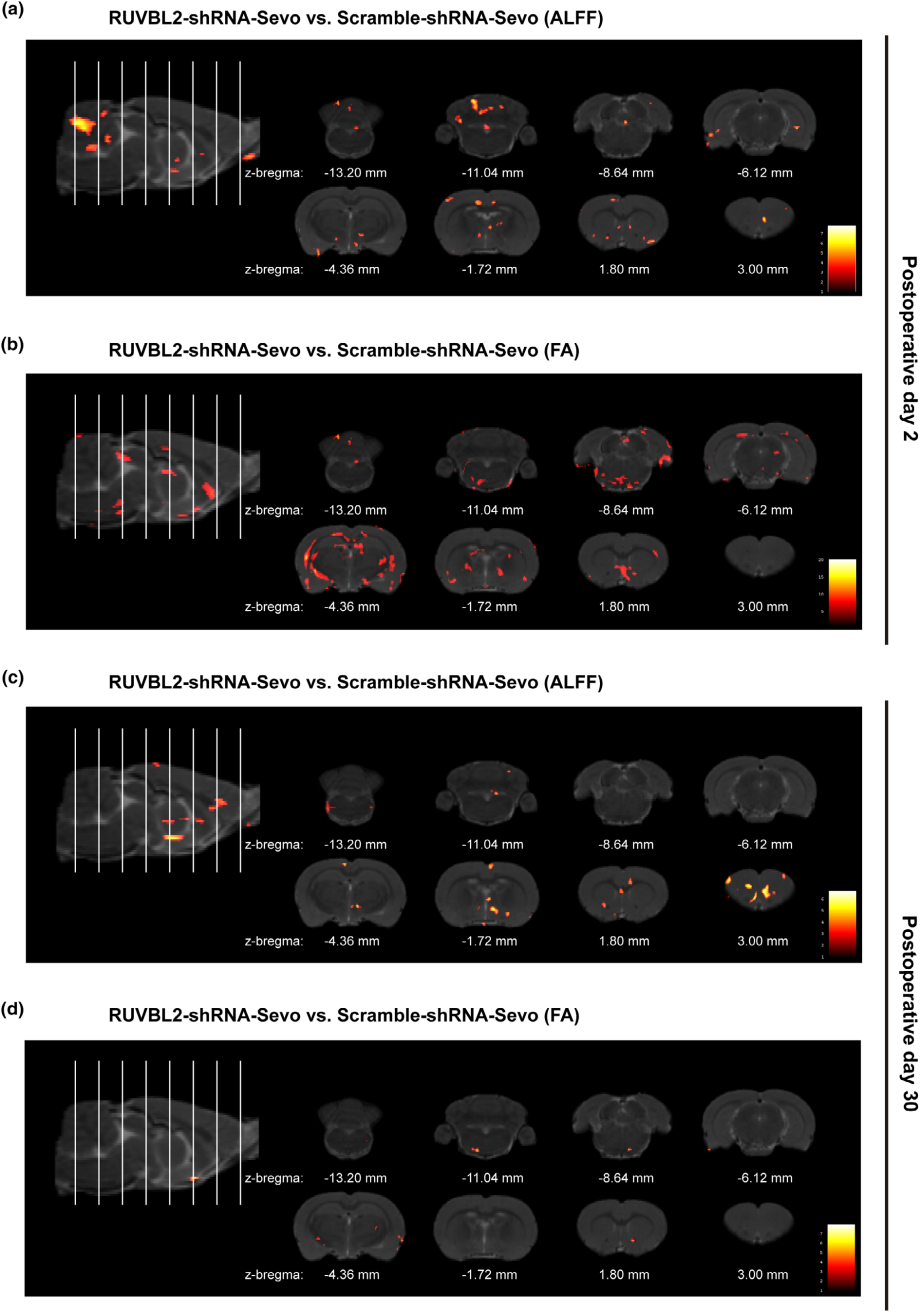
Functional magnetic resonance imaging (ALFF) results and diffusion tensor imaging (FA) of aged MCI rats. (a) Representative maps showing significant differences in ALFF values between the RUVBL2‐shRNA‐Sevo group and Scramble‐shRNA‐Sevo group on postoperative Day 2 (*n* = 3, two‐sample *t* test); (b) Representative maps showing significant differences in FA values between the RUVBL2‐shRNA‐Sevo group and Scramble‐shRNA‐Sevo group on postoperative Day 2 (*n* = 3, two‐sample *t* test); (c) Representative maps showing significant differences in ALFF values between the RUVBL2‐shRNA‐Sevo group and Scramble‐shRNA‐Sevo group on postoperative Day 30 (*n* = 3, two‐sample *t* test); (d) Representative maps showing significant differences in FA values between the RUVBL2‐shRNA‐Sevo group and Scramble‐shRNA‐Sevo group on postoperative Day 2 (*n* = 3, two‐sample *t* test). The white line on the sagittal image indicates the section of the corresponding representative coronal image. *p* < 0.01, cluster size >50. Warm colors indicate brain regions with enhanced functional activity.

Based on the above findings, we found that sevoflurane has the most significant impact on cognitive function in aged MCI rats, and knocking down RUVBL2 can improve these effects. Therefore, we performed fMRI and DTI scans on aged MCI rats subjected to sevoflurane anesthesia on postoperative Days 2 and 30. ALFF reflects changes in the blood oxygen level‐dependent (BOLD) signal, revealing spontaneous functional activity in the CNS (Xi et al., 2012). FA measures the anisotropic component of water diffusion, with higher values indicating better tissue organization and neural conductivity, reflecting the alignment and integrity of brain white matter tracts (Blamire, 2018). On postoperative Day 2, compared to the Scramble‐shRNA‐Sevo group, the aged MCI rats in RUVBL2‐shRNA‐Sevo group showed significantly increased ALFF in cingulate gyrus, striatum and other regions (Figure 5a, Table S3) and significantly increased FA in hippocampus and other regions (Figure 5b, Table S4). On postoperative Day 30, aged MCI rats in the RUVBL2‐shRNA‐Sevo group also exhibited significantly increased ALFF in retrosplenial cortex, thalamus‐medial nucleus group and other corresponding regions (Figure 5c, Table S5) and FA in thalamus‐lateral nucleus group and prefrontal cortex and other regions (Figure 5d, Table S6). We also compared the differences in ALFF and FA of the aged MCI rats between the Scramble‐shRNA‐Sevo group and the RUVBL2‐shRNA‐Sevo group at different postoperative time points. The results showed that both groups exhibited reduced ALFF and FA in certain brain regions on postoperative Day 2 compared to Day 30. However, it was observed that the reductions in the corresponding brain regions were less in the RUVBL2‐shRNA‐Sevo group compared to the Scramble‐shRNA‐Sevo group (Figure S5a–d, Tables S7–S10).

### 3.6 RUVBL2 does not modulate hnRNPA2B1‐SGs assembly

Several studies have shown that RUVBL2 depletion reduces the formation of SGs (Jain et al., 2016; Zaarur et al., 2015). This finding led us to consider whether RUVBL2 affects the assembly, phase transition, and disassembly of SGs by altering their dynamics. Compared with NC‐siRNA, all three independent RUVBL2‐siRNA oligos reduced the expression of RUVBL2 in rat primary hippocampal neurons, with RUVBL2‐siRNA‐#3 resulting in the highest knockdown efficiency (Figure S6a,b). RUVBL2‐siRNA‐#3 was used to knock down RUVBL2 in hypoxia‐exposed rat primary hippocampal neurons. Western blot analysis was performed to detect the expression level of RUVBL2 in neurons treated with sevoflurane in the NC‐siRNA group and the RUVBL2‐siRNA group. The results revealed that RUVBL2 expression was decreased in the RUVBL2‐siRNA group (Figure S6c,d). The neurons were exposed to sevoflurane and it was found that sevoflurane resulted in the formation of a greater number of hnRNPA2/B1‐SGs in both the NC‐siRNA group and the RUVBL2‐siRNA group (Figure S6e,f). However, there were no smaller hnRNPA2/B1‐SGs in hypoxia‐exposed neurons in the RUVBL2‐siRNA‐Sevo group than in the NC‐siRNA‐Sevo group (Figure S6g), indicating that RUVBL2 is not involved in the assembly of hnRNPA2/B1‐SGs.

### 3.7 Knockdown of RUVBL2 mediates the transition of hnRNPA2/B1‐SGs from the hydrogel phase to the liquid phase

**FIGURE 6:**
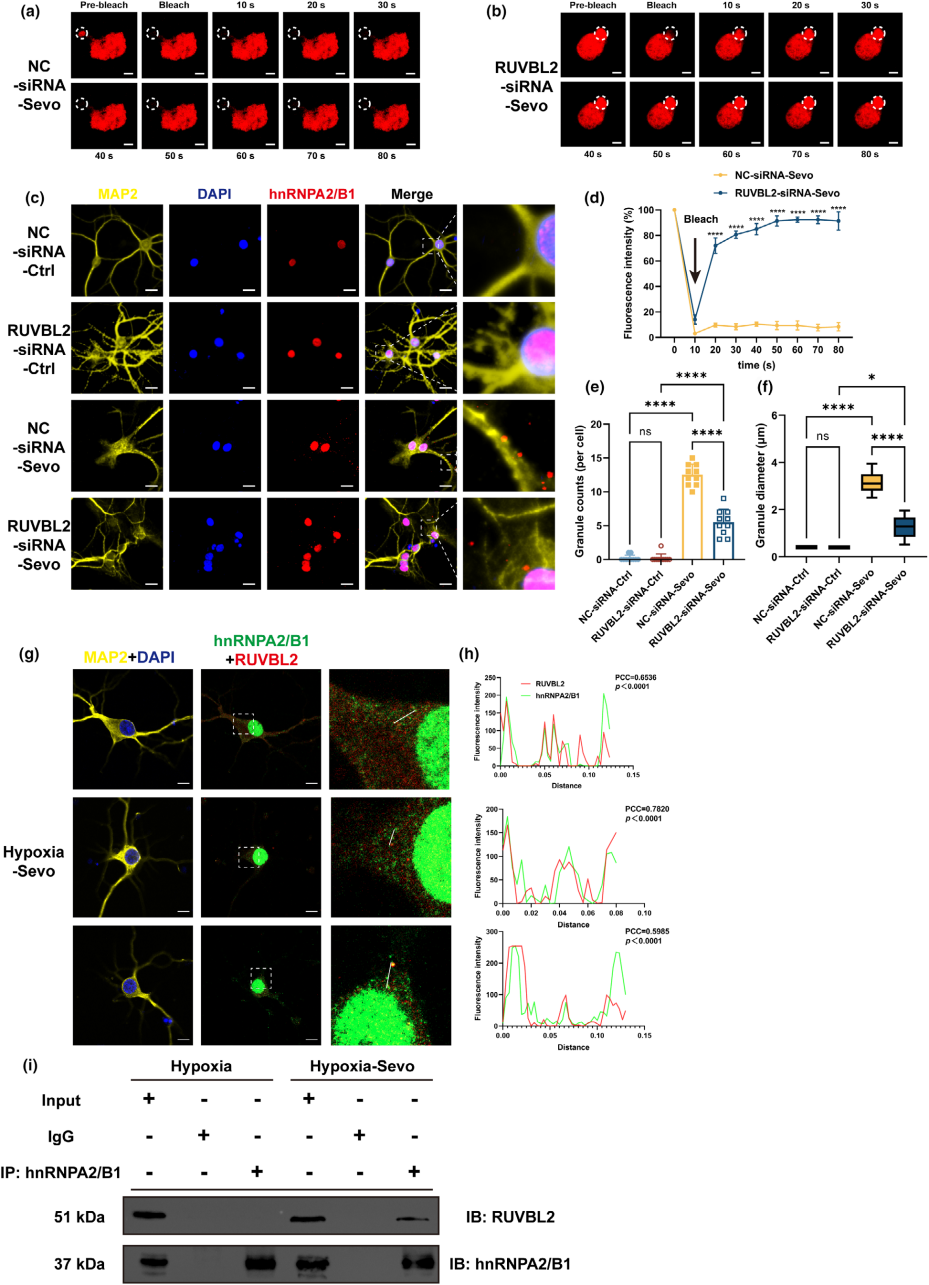
Knockdown of RUVBL2 mediates the transition of hnRNPA2/B1‐SGs from the hydrogel phase to the liquid phase and disassembly of hnRNPA2/B1‐SGs, and RUVBL2 co‐localizes and interacts with hnRNPA2/B1. (a, b) FRAP analysis of hnRNPA2/B1‐SGs formed in response to sevoflurane exposure in NC‐siRNA and RUVBL2‐siRNA rat primary hippocampal neurons. Scale bar = 3 μm. (d) Change in the fluorescence intensity in the ROI over time (*n* = 3 independent experiments, two‐way ANOVA followed by post hoc Bonferroni multiple comparisons test). (c) Disassembly dynamics of hnRNPA2/B1‐SGs in primary hippocampal neurons cultured in fresh medium for 1 h. Scale bar = 20 μm. (e, f) Statistical histogram of the counts and diameter of hnRNPA2/B1‐SGs (*n* = 10 independent experiments, one‐way ANOVA followed by post hoc Bonferroni multiple comparisons test). (g) Representative confocal images of hnRNPA2/B1 and RUVBL2 co‐localization. White lines indicate the ROI where fluorescence intensity profiles of hnRNPA2/B1 and RUVBL2 (Scale bar = 10 μm). (h) The fluorescence intensity distribution of hnRNPA2/B1 and RUVBL2 co‐localization within the ROI in (g) (PCC: Pearson's correlation coefficient). (i) Co‐immunoprecipitation of hnRNPA2/B1 and RUVBL2 after exposure to Hypoxia and Hypoxia‐Sevo. *p < 0.05, ****p < 0.0001; values are presented as the means ± SEMs.

SGs become less mobile as stress persists and display a range of forms from droplets to hydrogels to amyloid fibers (Schmidt & Gorlich, 2016). ATP maintains the exchangeable protein pool in SGs, facilitating their fluid‐like behavior (Dugger & Dickson, 2017). We co‐transfected rat primary hippocampal neurons with RUVBL2‐siRNA or NC‐siRNA and mCherry‐hnRNPA2/B1 plasmid. After exposure to sevoflurane, mCherry‐hnRNPA2/B1 was recruited to SGs, but the fluorescence intensity of mCherry‐hnRNPA2/B1 barely recovered to the initial level after photobleaching in the NC‐siRNA‐Sevo group (Figure 6a). Surprisingly, the fluorescence intensity of the resulting SGs recovered to a significantly greater extent after photobleaching in the RUVBL2‐siRNA‐Sevo group (Figure 6b,d). Taken together, these findings suggest that RUVBL2 alters the phase transition of SGs and facilitates the transition of SGs to the gel phase and even the fibrillary phase. Moreover, knocking down RUVBL2 reverses this process and renders SGs more fluid.

### 3.8 Effective clearance of SGs formed after sevoflurane exposure requires RUVBL2 knockdown

It was found that RUVBL2 knockdown promotes the transition of hnRNPA2/B1‐SGs to the liquid phase, leading us to hypothesize that RUVBL2 knockdown promotes the disassembly of hnRNPA2/B1‐SGs. To simulate the drug clearance in the brain, we administered sevoflurane to hypoxia‐exposed rat primary hippocampal neurons for 3 h and cultured the neurons with fresh Neurobasal medium containing B27 and L‐glutamine for 1 h. The hnRNPA2/B1‐SGs in the NC‐siRNA‐Sevo group were less than those in the RUVBL2‐siRNA‐Sevo group (Figure 6c,e). In neurons exposed to sevoflurane, RUVBL2 knockdown resulted in faster disassembly of hnRNPA2/B1‐SGs. This was consistent with the decrease in the diameter of SGs (Figure 6f). Therefore, abolishing RUVBL2 ATPase activity results in reduced protein interactions within SGs and quicker disassembly of SGs. This outcome indicates that RUVBL2 knockdown may decrease the persistence of SGs.

### 3.9 Sevoflurane affects ATP levels and the intracellular pH in hypoxia‐exposed neurons by interfering with RUVBL2

During cellular stress, the activity of the RUVBL1/2 complex is increased, and the complex is concentrated in SGs and inhibits the translation of mRNAs that regulate energy metabolism within SGs. Consequently, cellular energy metabolism is impaired (Chen, Hou, et al., 2022). The measurement of the ATP content in hypoxia‐exposed rat primary hippocampal neurons exposed to sevoflurane revealed that the ATP content was higher in RUVBL2‐knockdown neurons than in neurons without RUBVL2 knockdown (Figure S6d). These findings indicate that sevoflurane can impact neuronal energy metabolism. Abnormal energy metabolism disrupts the intracellular acid–base balance. The intracellular pH of hypoxia‐exposed rat primary hippocampal neurons in the RUVBL2‐siRNA and NC‐siRNA groups was analyzed using the BCECF AM probe. The results illustrated that the NC‐siRNA‐Sevo group neurons had weaker fluorescence than the RUVBL2‐siRNA‐Sevo group did, indicating a lower intracellular pH (Figure S6e).

### 3.10 RUVBL2 interacts with hnRNPA2/B1 to regulate hnRNPA2/B1‐SGs dynamics

Given that hnRNPA2/B1, as an RBP, translocates from the nucleus to the cytoplasm in response to stress, we investigated the interaction between hnRNPA2/B1 and RUVBL2 after exposure to sevoflurane. We confirmed that RUVBL2 co‐localization with hnRNPA2/B1 through immunofluorescence (Figure 6h,i). We conducted Co‐immunoprecipitation analysis of sevoflurane‐exposed hypoxia‐exposed neurons to confirm the direct interaction between RUVBL2 and hnRNPA2/B1. Firstly, the Western blot results showed that HIF‐1α expression in neurons exposed to hypoxia, as well as in hypoxia‐exposed neurons treated with sevoflurane, was higher than in neurons cultured under normoxic conditions (Figure S6j,k). As expected, Co‐immunoprecipitation with an anti‐RUVBL2 antibody resulted in the pulldown of hnRNPA2/B1 in the immune complex (Figure 6i). Taken together, our findings suggest that RUVBL2 interacts with hnRNPA2/B1 to contribute to the formation of hnRNPA2/B1‐SGs in response to sevoflurane exposure in hypoxia‐exposed rat primary hippocampal neurons.

## 4 DISCUSSION

The development of preventive and therapeutic approaches for PND has been hindered by numerous challenges, many of which stem from an incomplete understanding of its pathogenesis. In this study, we explored the role of anesthesia‐ and surgery‐induced hippocampal hnRNPA2/B1‐SGs formation in mediating PND development in aged MCI rats. We found that modulating hnRNPA2/B1‐SGs dynamics by knocking down RUVBL2, which affects energy metabolism, improved postoperative learning and memory function and functional activity in brain regions in aged MCI rats. The results of the present study demonstrated that RUVBL2 knockdown promotes the transition of hnRNPA2/B1‐SGs from the hydrogel phase to the liquid phase, accelerating their disassembly. These findings reveal the neuroprotective effect of RUVBL2 knockdown against the progression of PND, providing a potential therapeutic target for mitigating cognitive decline associated with anesthetics interventions in aged MCI patients.

Hydrophobic and aggregate‐prone protein inclusion bodies, the formation of which results from the abnormal accumulation of proteins, cause progressive disruption of neuronal function and are commonly found in neurodegenerative diseases, including Alzheimer's disease, Parkinson's disease, and amyotrophic lateral sclerosis (Dugger & Dickson, 2017). Pathological proteins such as P‐Tau, FUS and TDP‐43 have been found to co‐localize with SGs core proteins (Aulas et al., 2012; Jiang et al., 2021; Mandrioli et al., 2020; Szewczyk et al., 2023; Zhang et al., 2020). Although SGs have been extensively studied in various neurodegenerative diseases, research on the mechanisms underlying PND is lacking. Our previous research indicated that in aged MCI rats with PND after ORIF under sevoflurane anesthesia, hnRNPA2/B1 in the hippocampus is transported out of the nucleus and forms aggregates in the cytoplasm. This protein can be re‐translocated to the nucleus via Kap‐β2, thereby reducing the neurotoxicity of sevoflurane anesthesia (Zhang et al., 2023). However, Kap‐β2 appears to function more as a “remover” of hnRNPA2/B1 in this context. To address this, we focused on RUVBL2, an ATPase, in this study, aiming to regulate SGs dynamics at a relatively high level from an energy‐related perspective. Our findings indicate that in the hippocampus of aged MCI rats with PND, hnRNPA2/B1 aggregates are involved in the recruitment of SGs, whereas the knockout of RUVBL2 does not affect the assembly of hnRNPA2/B1‐SGs. These findings suggest that SGs accumulate in the cytoplasm of hippocampal neurons to facilitate pro‐survival adaptive responses when aged MCI rats are subjected to anesthetics and surgical stress. Moreover, RUVBL2 knockdown facilitates the transition of hnRNPA2/B1‐SGs from the hydrogel phase to the liquid phase. This results in a decrease in the binding of constituent proteins within the hnRNPA2/B1‐SGs. After stress dissipates, the accelerated disassembly of hnRNPA2/B1‐SGs ultimately exerts a neuroprotective effect, reducing the incidence of PND.

The phase transition of SGs is regulated mainly by their internal stability, which is affected by energy levels and pH. Wang and colleagues reported that inhibiting cellular glycolysis, which reduces ATP levels, delays the clearance of SGs. However, SGs clearance is restored following the overexpression of GLUT1 or supplementation with ATP‐containing liposomes, indicating that adequate glycolytic activity and an adequate ATP supply are essential for efficient SGs clearance (Wang et al., 2022). When ATP is depleted, the low‐complexity domains (LCDs) of SGs‐associated proteins likely transition from the droplet stage to the hydrogel or even the fibrillary phase. The core protein of SGs, G3BP1, also becomes enlarged and immobilized. These findings have implications for the pathological formation of SGs in various diseases (Jain et al., 2016; Kato et al., 2012). These findings also explain the elevated ATP content and the decrease in hnRNPA2/B1‐SGs mediated amyloid fibril formation in the hippocampus of aged MCI rats in the RUVBL2‐shRNA group after surgery. It has been demonstrated that in yeast and engineered proteins, a lower pH induces LLPS, promoting protein phase separation and the formation of membrane‐free organelles (Grimes et al., 2023; Liu et al., 2022). Our study revealed that RUVBL2 knockdown increased the pH of general anesthetic‐exposed rat primary hippocampal neurons. This knockdown makes hnRNPA2/B1‐SGs highly mobile and easily disassembled, reducing their propensity for pathological aggregates.

In aged MCI rats that were exposed to anesthesia and subjected to surgery, the co‐localization of hnRNPA2/B1 and TIA1 in the CA1 region of the hippocampus in the RUVBL2‐shRNA group progressively decreased from postoperative Days 2–30. In vitro, RUVBL2 knockdown increased the mobility and disassembly rate of hnRNPA2/B1‐SGs. We discovered that RUVBL2 binds to hnRNPA2/B1 during sevoflurane exposure. The absence of RUVBL2 activity may weaken the binding forces between hnRNPA2/B1 and other proteins, causing SGs to transition from the hydrogel phase to the liquid phase. This process likely increases ATP availability, thereby increasing SGs mobility by offsetting the binding of protein LCDs (Kato et al., 2012; Lin et al., 2015). Furthermore, the downregulation of RVB2, the yeast homologue of RUVBL2, reduces the proportion of cells forming large aggregate structures, leading instead to the formation of multiple smaller aggregates (Zaarur et al., 2015). Conversely, the deletion of some ATPases complexes, such as the Hsp70/Hsp40 and Cdc48/VCP complexes, results in more stable SGs (Buchan et al., 2013; Walters et al., 2015). Several AAA+ proteins, including Hsp104 and ClpB, function as molecular chaperones that facilitate the disassembly of SGs (Doyle & Wickner, 2009; Winkler et al., 2012). The variability in how different ATPase complexes affect SGs dynamics suggests that not all ATPases increase the stability of SGs. It is possible that these proteins function in distinct cellular compartments or are involved in different phases of SGs dynamics.

The RUVBL1/2 heterohexamer functions as an energy source within the R2TP complex, exerting ATPase activity (Zhao et al., 2005). However, the crystal structure of this heterohexamer indicates that hexamerization obstructs the nucleotide‐binding pocket, thereby hindering the conversion of ADP to ATP. This is mainly due to the N‐terminal region of the RUVBL1/2 complex blocking the nucleotide binding pocket (Munoz‐Hernandez et al., 2019; Silva et al., 2018; Yenerall et al., 2020). The DII domain within RUVBL1/2 plays a crucial role in the formation of stable heterohexamers and regulates both ATP hydrolysis and the deconjugase activity of the complex. Notably, DII‐truncated complexes exhibit greater ATPase and helicase activity than wild‐type complexes (Gorynia et al., 2011). Structurally, the RUVBL1/2 complex forms a heterohexamer with an internal channel, resembling that of typical chaperone proteins. The charge allocation within the channel facilitates interactions with single‐stranded DNA molecules, enabling the complex to exhibit DNA deconjugase activity. Consequently, it is plausible that the RUVBL1/2 complex aids in the assembling and maintenance of SGs by transporting polypeptide chains through the channel (Gorynia et al., 2011; Munoz‐Hernandez et al., 2019; Shen et al., 2000). These findings explain how RUVBL2 can influence SG dynamics through its functional and structural attributes when its levels are reduced.

Intravenous general anesthetics (e.g., propofol) and volatile general anesthetics (e.g., sevoflurane and desflurane) are the most commonly used agents in clinical practice. Under 3% sevoflurane anesthesia, rats can tolerate the stimuli caused by surgery (Chen, Qin, et al., 2022; Kashimoto et al., 1997). 9% desflurane was used because it has the same minimum alveolar concentration (MAC) as 3% sevoflurane (1.5 MAC) (Miller, 2015). The mean blood concentration of propofol used to maintain anesthesia is 4 μg·mL^−1^ (Sato et al., 2013). It was shown that when the mean arterial blood concentration of propofol in rats was 4, 1.2 and 0.6 μg·mL^−1^, the corresponding infusion rates were 40, 20 and 10 mg·kg^−1^·h^−1^, respectively (Logginidou et al., 2003). Therefore, we chose an infusion rate of 40 mg·kg^−1^·h^−1^ as the maintenance dose of propofol. This study compared the ability of sevoflurane, propofol and desflurane to promote the formation of hnRNPA2/B1‐SGs in the hippocampus of aged MCI rats to elucidate their roles in PND. Our findings indicated that sevoflurane promoted the formation of less mobile hnRNPA2/B1‐SGs than did propofol or desflurane, thereby affecting postoperative cognitive function in aged MCI rats. Clinical evidence supports that the occurrence of POD and dNCR in aged patients is lower when propofol anesthesia is used than when sevoflurane is used (Cao et al., 2023; Zhang et al., 2018), which is consistent with our mechanistic findings. An earlier study revealed no difference in the overall incidence of postoperative cognitive dysfunction between sevoflurane and desflurane anesthesia. However, patients anaesthetized with desflurane showed significant improvements in postoperative well‐being scale scores, recall of digit span scores, and scores in the Trail Making Test A (Rortgen et al., 2010). In vitro, sevoflurane resulted in the formation of more and larger hnRNPA2/B1‐SGs with lower mobility. Sevoflurane may directly interfere with mitochondrial metabolism, leading to NAD^+^ deficiency and insufficient ATP production, which impairs normal cellular functions (Zhu et al., 2022). Neurons exposed to sevoflurane exhibited a lower intraneuronal pH. The direct impact of sevoflurane on RUVBL2 expression disrupts cellular energy metabolism and pH homeostasis, thereby affecting SGs dynamics and exacerbating neuronal injury.

Despite the significant findings of this study regarding the role of RUVBL2 in influencing SGs dynamics and its pathophysiological mechanisms in aged MCI rats with PND, several limitations remain. First, proteomic analysis was not employed to identify novel RUVBL2‐interacting proteins or regulatory pathways that could contribute to understanding PND pathology, such as mitochondrial dysfunction, inflammation, or autophagy in aged MCI patients. Future research incorporating proteomics will provide a more comprehensive understanding of the molecular basis of RUVBL2 in PND and new targets for prevention and treatment. The second limitation of our study is the use of only male rats, which may overlook potential sex differences in PND development. We chose male rats to minimize variability related to hormonal fluctuations. Future studies should include both male and female rats to explore the potential influence of sex hormones on SGs dynamics, RUVBL2 function, and overall PND pathophysiology. This will provide a more comprehensive understanding and guide more personalized therapeutic approaches.

## 5 CONCLUSION

In summary, this study improves our understanding of the roles of RUVBL2 and hnRNPA2/B1‐SGs in PND. By demonstrating that anesthesia and surgery‐induced hnRNPA2/B1‐SGs mediate the occurrence of PND in aged MCI rats, this research highlights hnRNPA2/B1‐SGs as potential targets for preventing PND. Additionally, RUVBL2, as an ATPase, can regulate the dynamics of hnRNPA2/B1‐SGs and improve postoperative cognitive function and dynamic connectivity between brain regions in aged MCI rats. These findings suggest a novel and promising approach for delaying the progression to dementia due to PND in aged MCI patients.

## AUTHOR CONTRIBUTIONS

HYW, ZXW, and CYY conceived and designed the project, ZXW, XYW, MZ, HHL, XL, HL, and LZ conducted animal and in vitro experiments, ZXW, CYY, and XL analyzed data, ZXW and CYY wrote the manuscript.

## FUNDING INFORMATION

This work was supported by grants from the National Natural Science Foundation of China (No. 82371205 and 82071220); and Tianjin key Medical Discipline (Specialty) Construction Project (No. TJYXZDXK‐072C); and sponsored by Tianjin Health Research Project (No. TJWJ2023XK019).

## CONFLICT OF INTEREST STATEMENT

The authors declare that they have no competing interests.

## Supporting information


Data S1.

Data S2.

## Data Availability

The data that support the findings of this study are available from the corresponding author upon reasonable request.
