# Supplementary material for: Knockdown of RUVBL2 improves hnRNPA2/B1‐stress granules dynamics to inhibit perioperative neurocognitive disorders in aged mild cognitive impairment rats
Source: Aging Cell. 2024 Nov 28;24(3):e14418. doi: 10.1111/acel.14418 (PMC11896576; doi:10.1111/acel.14418)
Supplement: Supplementary file 1 — Data S1. [file ACEL-24-e14418-s001.pdf]

Supplementary Figures

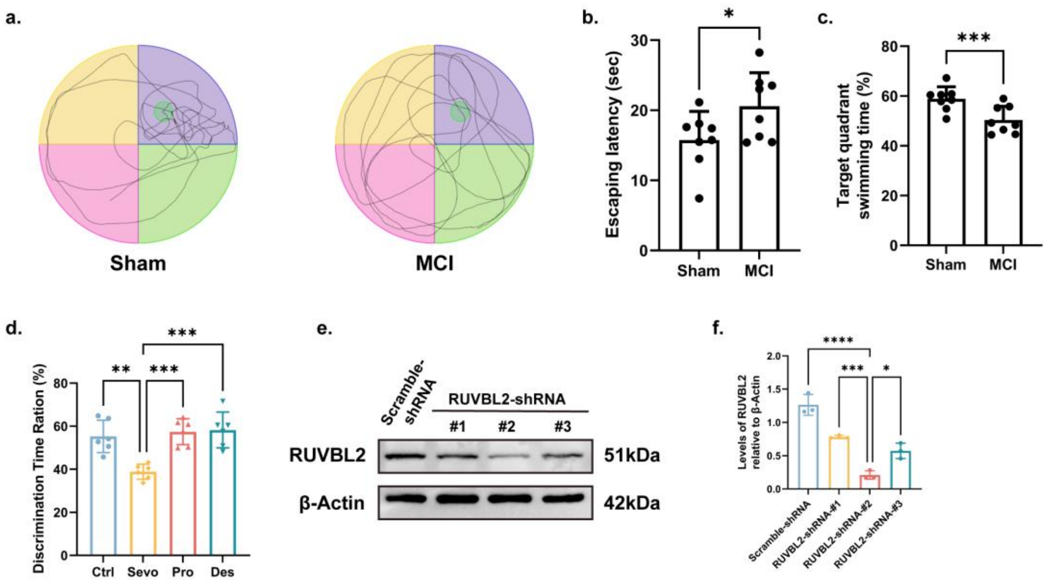

Figure S1 MCI screening and discrimination time ratio to novel object in the NOR test of aged MCI rats after surgery with diverse general anesthetics. a. Representative trajectory maps of water maze of rats in Sham and MCI groups. **b, c.** Escaping latency and target quadrant swimming time in MCI rats and Sham rats (n=8 independent experiments, unpaired *t* test). **d.** Discrimination time ratio for novel objects of aged MCI rats after anesthesia and surgery in the NOR test. (n=6 independent experiments, one-way ANOVA followed by post hoc Bonferroni multiple comparisons test). **e-f.** Representative western blot and statistical histogram of hippocampal lysates from Scramble-shRNA, RUVBL2-shRNA-#1, -#2 and -#3 aged MCI rats expressing RUVBL2. Western blots were labeled using to RUVBL2 and β-Actin antibodies (*n*=3 independent experiments, one-way ANOVA followed by post hoc Bonferroni multiple comparisons test). $^{*}P < 0.05$, $^{**}P < 0.01$, $^{***}P < 0.001$ and $^{****}P < 0.0001$. Values are expressed as mean ± SEM.

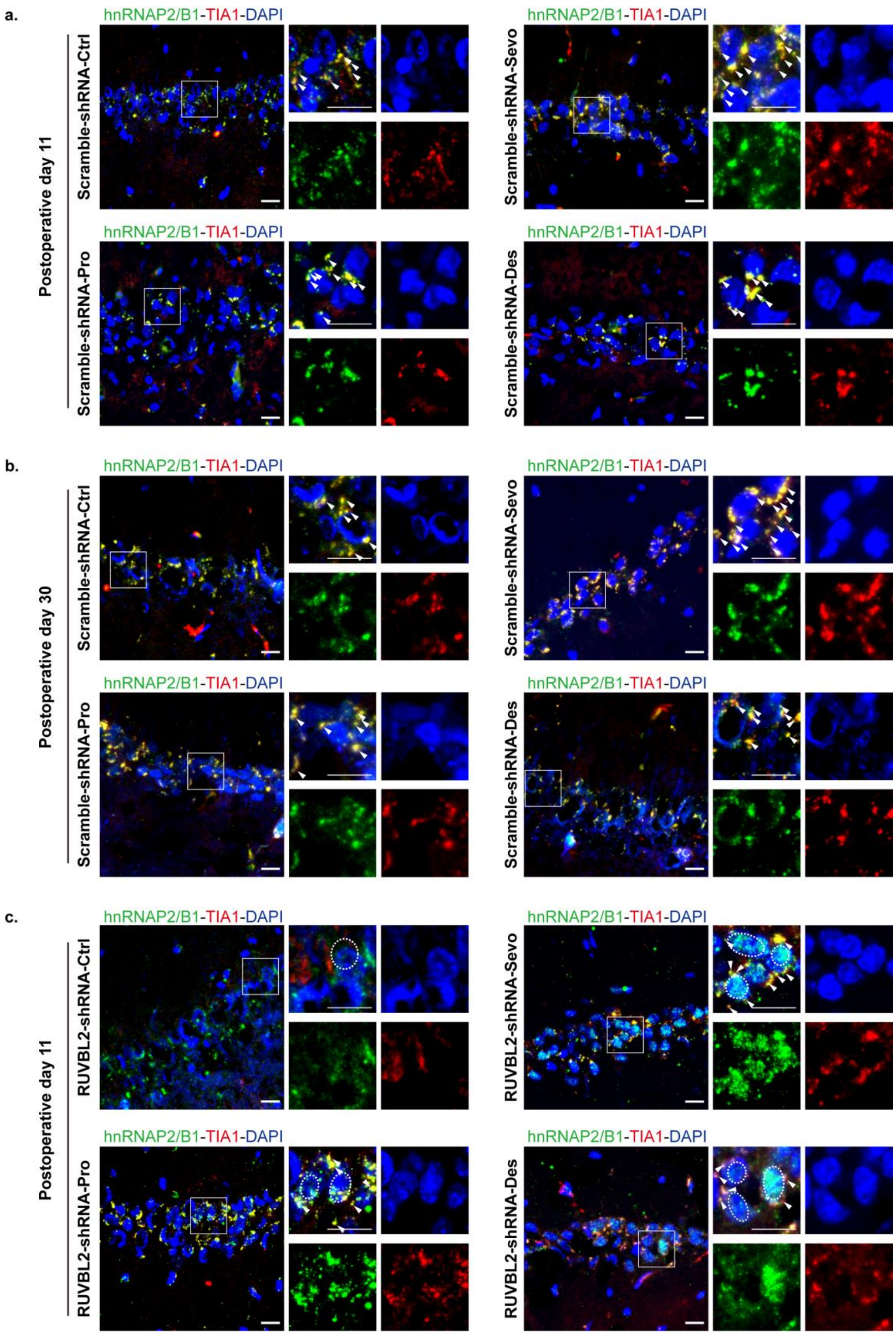

Figure S2 hnRNP2/B1-SGs in hippocampal CA1 of aged MCI rats in the Scramble-shRNA group still exist on post-anesthesia and surgery day 11 and day 30 , whereas RUVBL2 knockdown on day 11 returned hnRNP2/B1 to the nucleus. a. Confocal microscopy

representative images of hnRNPA2/B1 and TIA1 expression in the hippocampal CA1 region of the Scramble-shRNA group of aged MCI rats post-anesthesia and surgery day 11. **b.** Confocal microscopy representative images of hnRNPA2/B1 and TIA1 expression in the hippocampal CA1 region of the Scramble-shRNA group of aged MCI rats post-anesthesia and surgery day 30. **c.** Confocal microscopy representative images of hnRNPA2/B1 and TIA1 expression in the hippocampal CA1 region of the RUVBL2-shRNA group of aged MCI rats post-anesthesia and surgery day 30. Arrows: the regions in which hnRNPA2/B1 and TIA1 were colocalized. Dashed circle: the regions in which hnRNPA2/B1 translocated to the neclus. Scale bar = 20 μm.

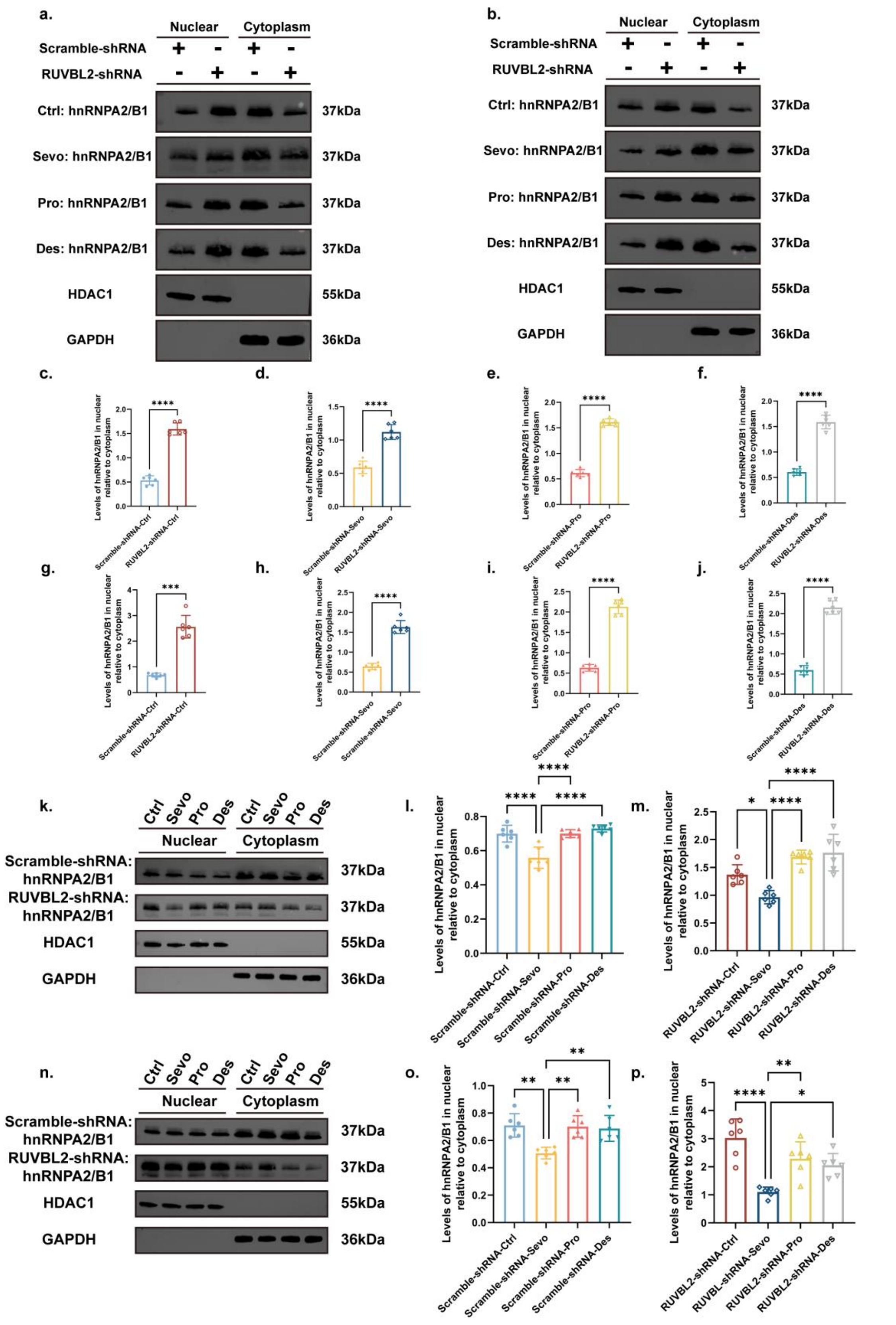

Figure S3 RUVBL2 knockdown enhances hnRNPA2/B1 nucleoplasmic ratio on post-anesthesia and surgery day 11 and day 30. a, c-f. Representative western blot and statistical histogram of

hippocampal nuclei and cytoplasmic lysates expressing hnRNPA2/B1 from aged MCI rats post-sevoflurane-, propofol-, desflurane-anesthetized and surgery day 11. Western blots were labeled using to hnRNPA2/B1 HDAC1 and GAPDH antibodies. HDAC1 as a nuclei internal reference protein and GAPDH as a cytoplasmic internal reference protein (n=6 independent experiments, unpaired t-test). **b, g-j.** Representative western blot and statistical histogram of hippocampal nuclei and cytoplasmic lysates expressing hnRNPA2/B1 from aged MCI rats post-sevoflurane-, propofol-, desflurane-anesthetized and surgery day 30. Western blots were labeled using to hnRNPA2/B1 HDAC1 and GAPDH antibodies. HDAC1 as a nuclei internal reference protein and GAPDH as a cytoplasmic internal reference protein (n=6 independent experiments, unpaired t-test). **k-m.** Representative western blot and statistical histogram of hippocampal nuclei and cytoplasmic lysates expressing hnRNPA2/B1 from aged MCI rats post-sevoflurane-, propofol-, desflurane-anesthetized and surgery day 11. Western blots were labeled using to hnRNPA2/B1, HDAC1 and GAPDH antibodies. HDAC1 as a nuclei internal reference protein and GAPDH as a cytoplasmic internal reference protein (n=6 independent experiments, one-way ANOVA followed by post hoc Bonferroni multiple comparisons test). **n-p.** Representative western blot and statistical histogram of hippocampal nuclei and cytoplasmic lysates expressing hnRNPA2/B1 from aged MCI rats post-sevoflurane-, propofol-, desflurane-anesthetized and surgery day 30. Western blots were labeled using to hnRNPA2/B1, HDAC1 and GAPDH antibodies. HDAC1 as a nuclei internal reference protein and GAPDH as a cytoplasmic internal reference protein (n=6 independent experiments, one-way ANOVA followed by post hoc Bonferroni multiple comparisons test). $^{*}P < 0.05$, $^{**}P < 0.01$, $^{***}P < 0.001$ and $^{****}P < 0.0001$. Values are expressed as mean ± SEM.

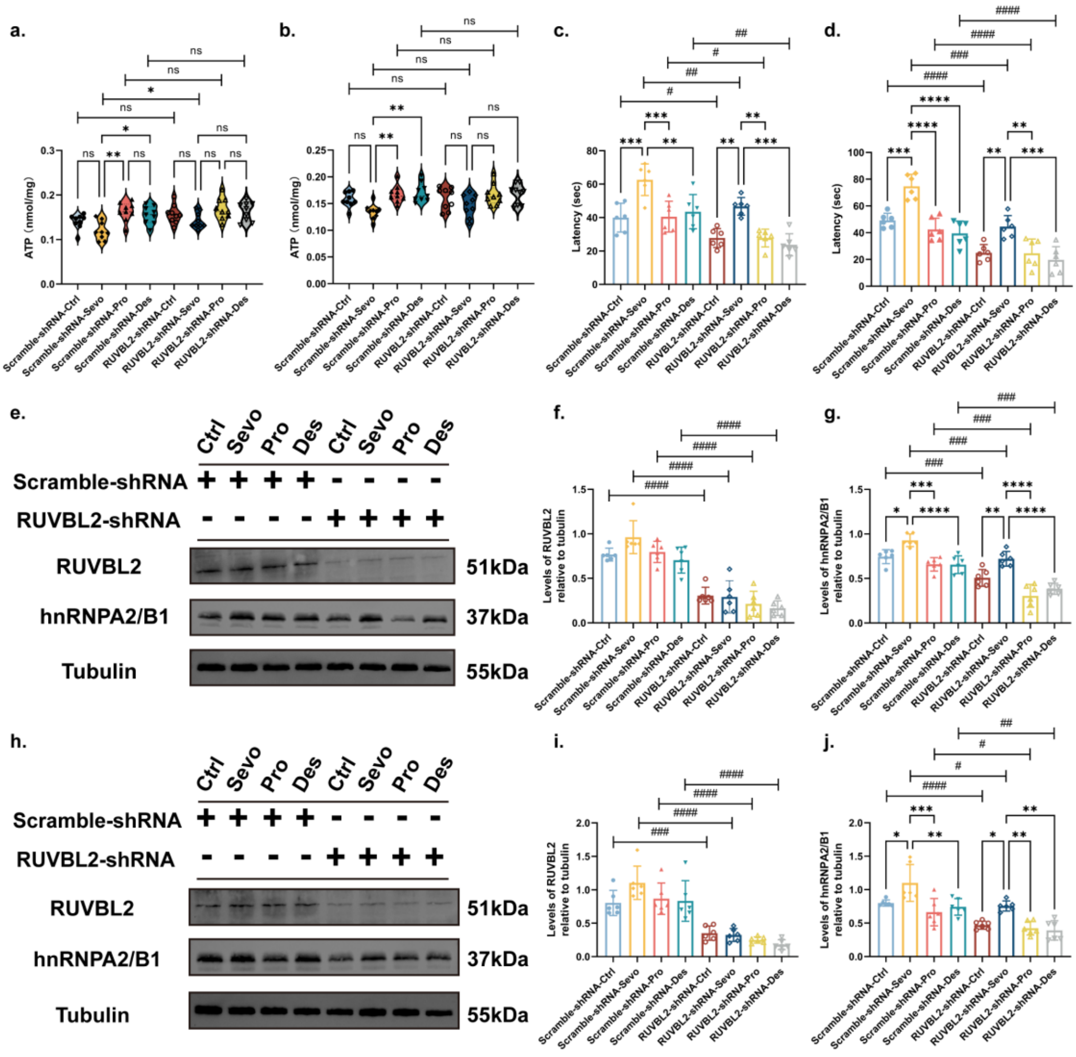

Figure S4 RUVBL2 knockdown increases ATP levels and improves learning and memory in MCI rats after anesthesia and surgery. a. ATP levels in the hippocampus of aged MCI rats at post-anesthesia and surgery day 11 (*n*=9 independent experiments, unpaired *t*-test and one-way ANOVA followed by post hoc Bonferroni multiple comparisons test). **b.** ATP levels in the hippocampus of aged MCI rats at post-anesthesia and surgery day 30 (*n*=9 independent experiments, unpaired *t*-test and one-way ANOVA followed by post hoc Bonferroni multiple comparisons test). **c.** Latency for target hole in the Barnes maze test of aged MCI rats on post-anesthesia and surgery day 11 (*n*=6 independent experiments, unpaired *t*-test and one-way ANOVA followed by post hoc Bonferroni multiple comparisons test). **d.** Latency for target hole in the Barnes maze test of aged MCI rats on post-anesthesia and surgery day 30 (*n*=6 independent experiments, unpaired *t*-test and one-way ANOVA followed by post hoc Bonferroni multiple comparisons test). **e-g.** Representative western blot and statistical histogram of hippocampal lysates from aged MCI rats expressing hnRNPA2/B1 and RUVBL2 on post-anesthesia and surgery day

11. Western blots were labeled using to hnRNPA2/B1, RUVBL2 and β-tubulin antibodies (*n*=6 independent experiments, unpaired *t*-test and one-way ANOVA followed by post hoc Bonferroni multiple comparisons test). **h-j.** Representative western blot and statistical histogram of hippocampal lysates from aged MCI rats expressing hnRNPA2/B1 and RUVBL2 on post-anesthesia and surgery day 30. Western blots were labeled using to hnRNPA2/B1, RUVBL2 and β-tubulin antibodies (*n*=6 independent experiments, unpaired *t*-test and one-way ANOVA followed by post hoc Bonferroni multiple comparisons test). $^{*}P < 0.05$, $^{**}P < 0.01$, $^{***}P < 0.001$ and $^{****}P < 0.0001$. $^{\#}P < 0.05$, $^{\#\#}P < 0.01$, $^{\#\#\#}P < 0.001$ and $^{\#\#\#\#}P < 0.0001$. Values are expressed as mean ± SEM.

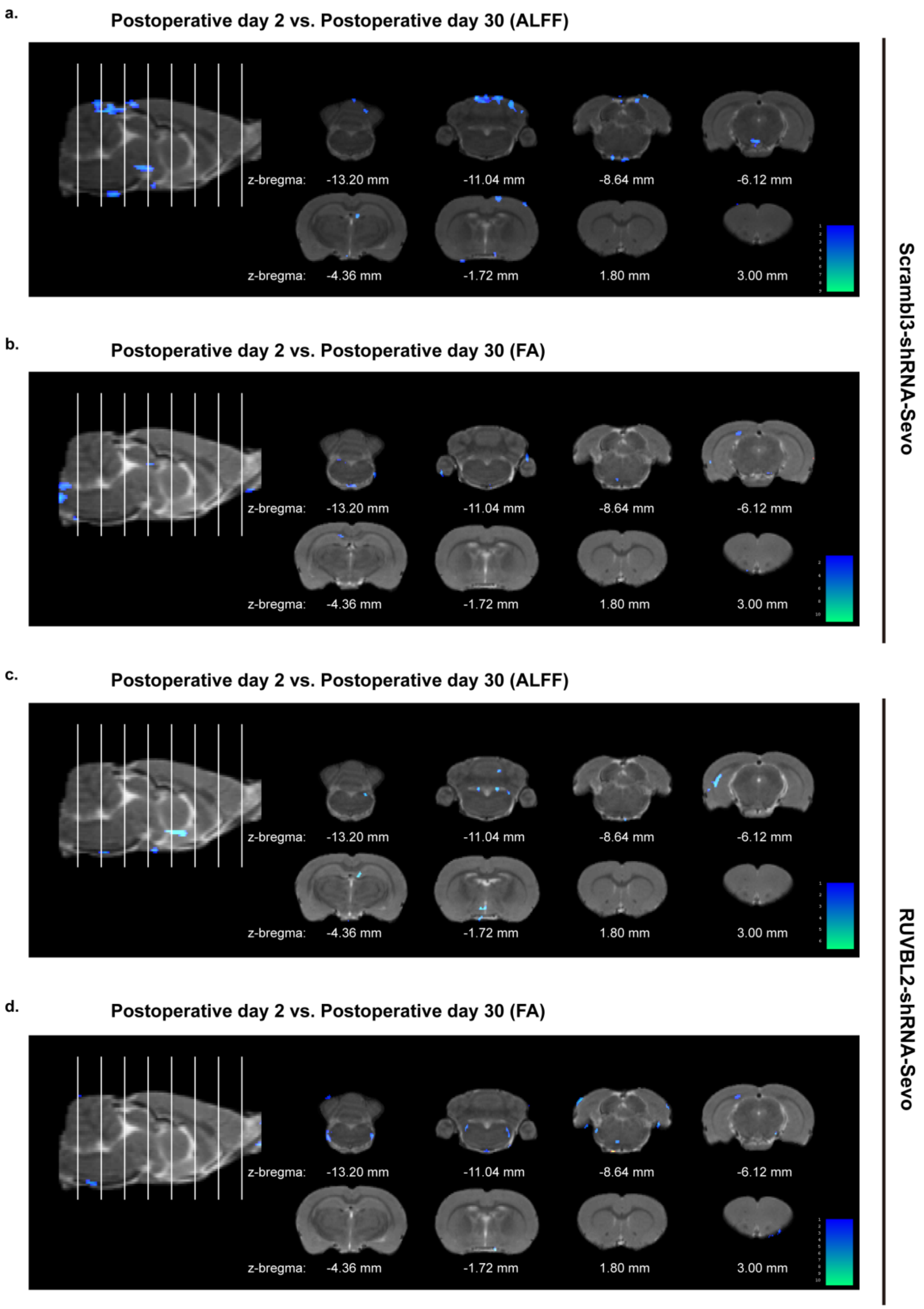

Figure S5 Functional magnetic resonance imaging (ALFF) results and diffusion tensor imaging (FA) of aged MCI rats. a. Representative maps showing significant differences in ALFF values between postoperative day 2 and postoperative day 30 in Scramble-shRNA-Sevo group (n=3, two-sample *t* test);

b. Representative maps showing significant differences in FA values between postoperative day 2 and postoperative day 30 in Scramble-shRNA-Sevo group (n=3, two-sample *t* test); **c.** Representative maps showing significant differences in ALFF values between postoperative day 2 and postoperative day 30 in RUVBL2-shRNA-Sevo group (n=3, two-sample *t* test); **d.** Representative maps showing significant differences in FA values between postoperative day 2 and postoperative day 30 in RUVBL2-shRNA-Sevo group (n=3, two-sample *t* test). The white line on the sagittal image indicates the section of the corresponding representative coronal images. $P < 0.01$, cluster size > 50. Cool colors indicate brain regions with diminished functional activity.

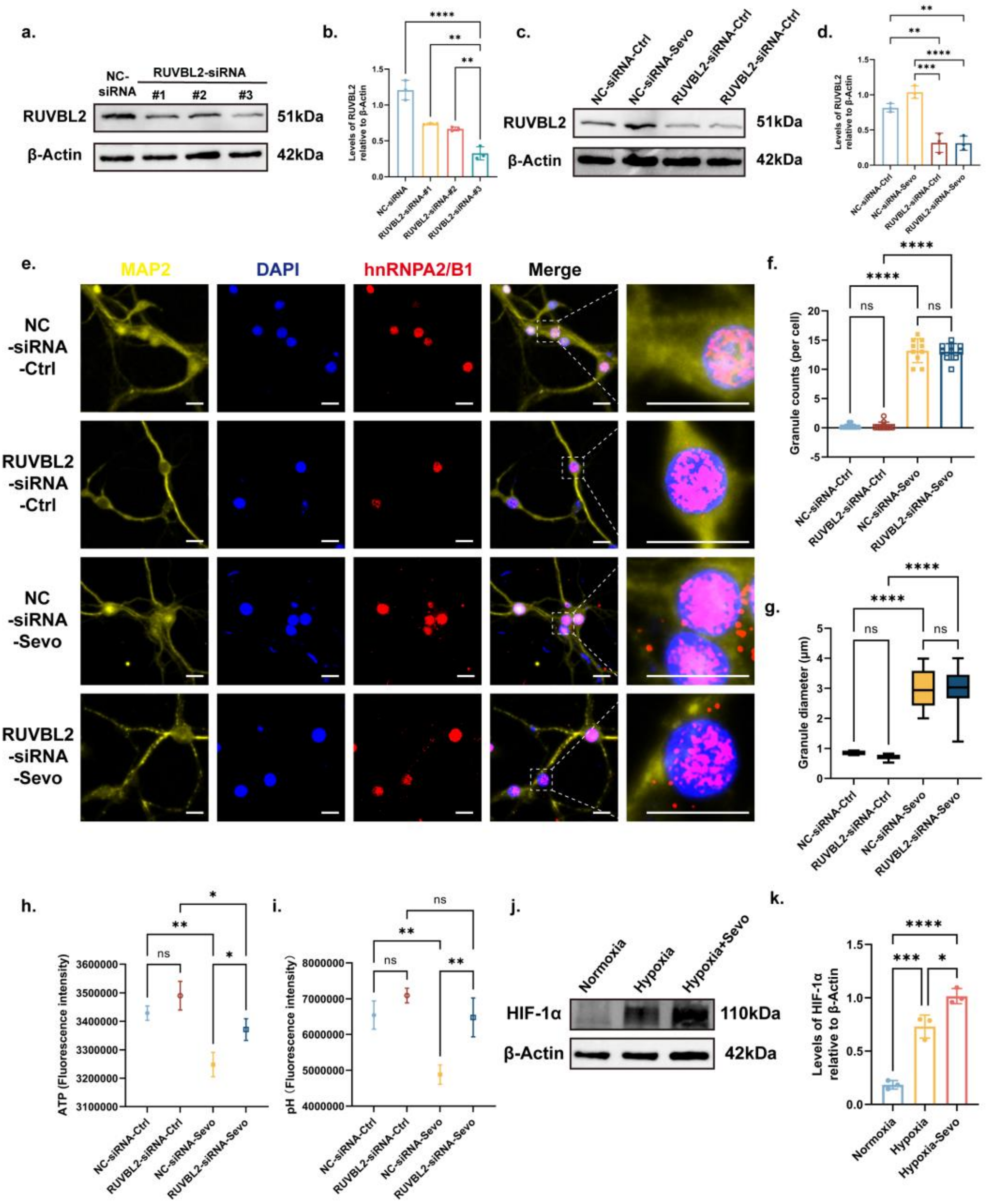

Figure S6 Knocking down RUVBL2 does not modulate the assembly of hnRNPA2B1-SGs and sevoflurane impact ATP levels and intracellular pH in hypoxia-exposed neurons by interfering with RUVBL2. a, b. Representative western blot and statistical histogram of rat primary hippocampal neurons from NC-siRNA, RUVBL2-siRNA-#1, -#2 and -#3 expressing RUVBL2. Western blots were labeled using to RUVBL2 and β-Actin antibodies (*n*=3 independent experiments, one-way ANOVA followed by post hoc Bonferroni multiple comparisons test). **c, d.** Representative western blot and statistical histogram of rat primary hippocampal neurons from NC-siRNA-Ctrl, NC-siRNA-Sevo, RUVBL2-siRNA-Ctrl, and RUVBL2-siRNA-Sevo expressing RUVBL2. Western blots were labeled using to RUVBL2 and β-Actin antibodies (*n*=3 independent experiments, one-way ANOVA followed

by post hoc Bonferroni multiple comparisons test). **e.** Assembly dynamics of hnRNPA2/B1-SGs in rat primary hippocampal neurons exposed to sevoflurane for 3 hours. Scale bar = 20 μm. **f, g.** The granules counts and diameter of hnRNPA2/B1-SGs (n=10 independent experiments, one-way ANOVA followed by post hoc Bonferroni multiple comparisons test). **h.** Fluorescence intensity of the ATP probe in rat primary hippocampal neurons after 3 hours of control, sevoflurane exposure (n=3 independent experiments, one-way ANOVA followed by post hoc Bonferroni multiple comparisons test). **i.** Fluorescence intensity of the pH probe in rat primary hippocampal neurons after 3 hours of control, sevoflurane exposure (n=3 independent experiments, one-way ANOVA followed by post hoc Bonferroni multiple comparisons test). **j, k.** Representative western blot and statistical histogram of rat primary hippocampal neurons from Normoxia, Hypoxia and Hypoxia-Sevo expressing HIF-1α. Western blots were labeled using to HIF-1α and β-Actin antibodies (n=3 independent experiments, one-way ANOVA followed by post hoc Bonferroni multiple comparisons test). *$P < 0.05$, **$P < 0.01$, ***$P < 0.001$ and ****$P < 0.0001$, Values are expressed as mean ± SEM.
