# Supplementary material for: Knockdown of RUVBL2 improves hnRNPA2/B1‐stress granules dynamics to inhibit perioperative neurocognitive disorders in aged mild cognitive impairment rats
Source: Aging Cell. 2024 Nov 28;24(3):e14418. doi: 10.1111/acel.14418 (PMC11896576; doi:10.1111/acel.14418)
Supplement: Supplementary file 2 — Data S2. [file ACEL-24-e14418-s002.pdf]

Supplementary Tables

Table S1 shRNA sequences targeting rat RUVBL2

Gene ID	Sequence
**RUVBL2 #1**	5’-GGA GAC AGA GAT CAT TGA AGG TTC AAG AGA CCT TCA ATG ATC TCT GTC TCC-3’
**RUVBL2 #2**	5’-GGA GGA GAC AGA GAT CAT TGA TTC AAG AGA TCA ATG ATC TCT GTC TCC TCC-3’
**RUVBL2 #3**	5’-GAT GGA GAC CAT CTA CGA TCT TTC AAG AGA AGA TCG TAG ATG GTC TCC ATC-3’

Table S2 siRNA sequences targeting rat RUVBL2

Gene ID	Sequence
**RUVBL2 #1**	5’-GAG AGU UUC UCU UUC CUA ATT-3’
	5’-UUA GGA AAG AGA AAC UCU CTT-3’
**RUVBL2 #2**	5’-GGA GAC AGA GAU CAU UGA ATT-3’
	5’-UUC AAU GAU CUC UGU CUC CTT-3’
**RUVBL2 #3**	5’-GCC CGA GAC UAU GAU GCC ATT-3’
	5’-UGG CAU CAU AGU CUC GGG CTT-3’

Table S3 Brain regions showing abnormal ALFF in aged MCI rats

Brain regions	Ke	t value	MNI coordinates		
			X	Y	Z
**Postoperative day 2**					
**RUVBL2-shRNA-Sevo > Scramble-shRNA-Sevo**					
cingulate gyrus _right	79	6.4247	0.8378	6.2563	-13.3179
visual cortex _right	79	5.4305	4.8908	5.7157	-6.1179
striatum_right	77	4.5537	3.7126	7.3085	1.5621
cerebellum _poaterior lobe of cerebellum_right	72	6.5084	0.8378	6.1147	-13.3179
sensory cortex _left	72	5.1402	-3.6001	4.6627	-11.3979
retrosplenial cortex_right	65	5.5252	0.3341	3.4969	4.4421
amygdaloid body_left	63	5.2378	-4.5015	9.3336	-3.7179
cerebellum _poaterior lobe of cerebellum_right	51	4.1917	1.8772	4.6346	-0.8379
cerebellum _anterior lobe of cerebellum _left	50	4.8673	-0.3955	4.6219	-10.4379
insular cortex_left	48	6.4928	-5.3674	7.4944	0.1221
olfactory bulb_left	48	5.4433	-0.9033	4.8672	6.3621
midbrain _ superior colliculus _left	44	5.3155	-0.5054	5.0318	6.3621
pontine _ tegmentum of pons_left	39	4.2625	-2.0112	8.9982	0.1221
cerebellum _anterior lobe of cerebellum _left	39	5.0525	-4.051	6.0319	2.5221
cingulate gyrus _right	37	5.167	0.2421	4.9569	-8.9979
cerebellum _anterior lobe of cerebellum _right	36	4.8164	0.2487	5.1588	-9.4779
auditory cortex_left	35	4.7108	-6.7282	6.2545	-6.5979
cingulate gyrus _left	32	4.3207	-0.1162	5.7736	-11.8779
pontine _ basilar part of pons _right	30	4.8429	3.4292	7.3915	2.0421
cerebellum _anterior lobe of cerebellum _right	27	4.5666	3.3147	7.4271	1.5621
cerebellum _poaterior lobe of cerebellum_right	27	4.1426	0.2749	5.4515	-1.3179
sensory cortex _left	26	4.7941	-4.1828	5.9249	2.0421
corpus callosum _right	26	3.9376	1.3541	5.1313	-1.7979
insular cortex_right	25	4.7997	3.8444	7.2739	2.0421
cerebellum _poaterior lobe of cerebellum_left	24	4.1684	-0.6395	7.2776	-2.7579
cerebellum _anterior lobe of cerebellum _left	24	3.8535	-0.8373	5.1339	1.5621
hippocampus_right	24	6.1048	2.594	5.7817	-5.1579
Thalamus_medial nucleus group_left	23	4.4045	-0.5011	7.4449	-2.7579
pfi flocculonodular lobe _left	22	4.6559	-5.7653	7.613	0.1221
motor cortex _right	22	4.4754	1.8706	4.7336	-0.3579
retrosplenial cortex_left	22	4.274	-0.8503	4.6255	-7.5579
medulla oblongata_right	21	4.2478	2.2619	7.4929	0.1221
insular cortex_left	21	4.4916	-3.6597	6.4548	3.0021
cerebellum _poaterior lobe of cerebellum_left	21	4.8582	-3.7517	5.012	-10.4379
cerebellum _poaterior lobe of cerebellum_left	20	4.1216	-1.1956	6.2515	-1.3179
cingulate gyrus _left	20	4.1166	-0.8373	4.9923	1.5621
corpus callosum _right	20	5.3601	0.9762	6.2643	-13.3179
pontine _ tegmentum of pons_right	18	4.2324	1.7322	9.2568	-0.3579
cerebellum _poaterior lobe of cerebellum_right	18	4.0259	2.4069	6.9594	-0.3579
cingulate gyrus _right	18	4.3894	0.2749	5.3099	-1.3179
third ventricle_left	17	4.2312	-4.5015	8.7495	-3.7179
striatum_left	17	4.2621	-1.8901	8.8636	0.1221

olfactory cortex_left	17	4.0342	-7.2711	7.6323	-6.1179
motor cortex _right	17	4.424	0.4552	3.9464	4.4421
striatum_left	16	4.4494	-2.4528	6.3258	2.0421
Thalamus_medial nucleus group_right	16	4.3665	1.5016	6.7601	-3.7179
cerebellum _anterior lobe of cerebellum _left	16	4.0921	-0.1228	5.5894	-11.3979
claustral layer _left	15	6.3044	-5.229	7.5024	0.1221
pfi flocculonodular lobe _right	15	4.5899	3.4358	7.4341	1.5621
striatum_right	15	3.808	2.5016	6.6544	1.5621
cerebellum _anterior lobe of cerebellum _right	15	4.4495	0.4267	6.0065	-12.3579
medulla oblongata_left	14	4.0113	-0.6395	7.5785	-2.7579
dentate gyrus_right	14	6.0471	2.5874	5.7391	-4.6779
cerebellum _cerebellar nucleus_right	14	3.9755	1.4752	5.2799	-1.7979
hypothalamus_preoptic region_right	13	3.9149	1.7322	9.1152	-0.3579
Thalamus_lateral nucleus group_right	13	4.1107	0.5674	7.8743	-3.7179
fourth ventricle _right	13	4.3386	2.2685	7.2346	-0.3579
pontine _ tegmentum of pons_right	13	4.5352	3.1697	7.3765	2.0421
temporal association cortex_left	13	3.9922	-7.15	7.3561	-6.1179
cerebellum _anterior lobe of cerebellum _left	13	4.3749	-2.4528	6.4674	2.0421
frontal association cortex_left	13	4.0865	-0.6199	4.9258	5.8821
auditory cortex_left	13	3.9732	-7.038	5.4414	-4.1979
cerebellum _anterior lobe of cerebellum _right	13	4.3101	0.6266	4.8931	2.0421
corpus callosum _right	13	5.422	4.8842	5.6731	-5.6379
cingulate gyrus _right	12	4.2424	0.6332	5.0773	1.5621
cerebellum _poaterior lobe of cerebellum_left	12	5.4844	-0.8922	6.0147	-13.3179
cingulate gyrus _left	12	4.2118	-0.1426	5.0191	-9.9579
Thalamus_lateral nucleus group_left	11	4.3094	-1.9584	7.1442	-3.7179
anterior nucleus group _left	11	3.9274	-1.3274	6.4277	-1.7979
retrosplenial cortex_right	11	3.9034	0.2815	5.3525	-1.7979
striatum_right	10	4.3508	3.9853	8.276	0.6021
Thalamus_lateral nucleus group_left	10	4.2144	-3.9586	7.6549	-4.1979
cerebellum _poaterior lobe of cerebellum_left	10	4.0861	-0.2546	5.624	-11.8779
visual cortex _right	10	4.5214	1.4948	2.7688	6.8421
hypothalamus_preoptic region_left	9	4.2833	-2.0046	8.8992	-0.3579
corpus callosum _right	9	4.6121	3.851	7.3165	1.5621
cerebellum _anterior lobe of cerebellum _right	9	3.9694	2.5016	6.796	1.5621
hippocampus_left	9	4.6548	-1.0417	4.6999	6.3621
hippocampus_right	9	4.185	1.6136	5.4295	-1.7979
parietal cortex posterior area_right	9	5.1449	6.3547	5.174	-5.6379
capsule_right	8	3.8511	3.3807	8.5788	-3.2379
cerebral peduncle _left	8	4.481	-3.8375	7.9628	-4.1979
claustral layer _right	8	4.7824	3.706	7.2659	2.0421
retrosplenial cortex_left	8	4.4331	-0.9033	4.7079	6.3621
motor cortex _left	8	3.6715	-0.8503	4.7671	-7.5579
cerebellum _anterior lobe of cerebellum _left	7	4.2106	-5.3806	6.9667	1.0821
cerebellum _anterior lobe of cerebellum _right	7	4.2579	-2.4462	6.51	1.5621
septal area _left	7	3.6233	-1.2022	6.3505	-0.8379
olfactory bulb_right	7	4.9586	2.1829	5.5319	-4.1979
cerebellum _anterior lobe of cerebellum _right	7	3.98	0.2683	5.1257	-0.8379
cerebellum _anterior lobe of cerebellum _left	6	3.7184	-1.334	6.5267	-1.3179

Thalamus_medial nucleus group_left	6	3.7081	-0.1098	6.3987	-2.2779
cerebellum _poaterior lobe of cerebellum_right	6	3.6277	0.0113	6.4057	-2.2779
orbital cortex_left	6	4.8309	-0.4988	5.216	5.8821
PFC_left	6	4.8309	-0.4988	5.216	5.8821
sensory cortex _right	6	4.5048	1.8772	4.7762	-0.8379
cingulate gyrus _left	6	4.8113	-0.8922	5.8554	-13.3179
corpus callosum _left	6	4.5074	-1.0306	5.8474	-13.3179
striatum_right	5	3.5605	3.4886	8.5006	-2.2779
hypothalamus_tuberal region _right	5	4.0191	0.7058	8.1832	-3.7179
medulla oblongata_right	5	3.6549	0.7058	8.3248	-3.7179
anterior nucleus group _left	5	3.88	-0.6461	7.3943	-2.2779
corpus callosum _left	5	4.1593	-5.8006	5.6818	-6.1179
cerebellum _anterior lobe of cerebellum _right	5	4.1546	1.8706	4.8752	-0.3579
cingulate gyrus _right	5	3.9471	1.3607	4.873	-2.2779
hippocampus_right	5	4.0867	4.8908	5.9989	-6.1179
blood vessel _left	5	4.1864	0.2089	3.2732	3.4821
cerebellum _anterior lobe of cerebellum _right	5	3.7579	0.2287	3.401	2.0421
frontal association cortex_right	5	3.8833	0.7188	4.4613	5.4021
cerebellum _anterior lobe of cerebellum _left	5	4.1436	-0.446	3.362	2.0421
retrosplenial cortex_right	5	4.1369	1.3564	2.7608	6.8421
cerebellum _poaterior lobe of cerebellum_right	4	3.8741	0.5608	7.9733	-3.2379
claustral layer _left	4	4.2383	-3.6531	6.4974	2.5221
pontine _ tegmentum of pons_left	4	4.6405	-3.5213	6.6044	3.0021
Thalamus_medial nucleus group_left	4	3.6461	-1.3274	6.7286	-1.7979
third ventricle_left	4	3.9995	-1.1956	6.3931	-1.3179
temporal association cortex_left	4	4.0605	-6.86	6.2891	-7.0779
insular cortex_left	4	4.8839	-0.6265	5.0248	6.3621
parietal cortex posterior area_left	4	4.3246	-5.939	5.6738	-6.1179
visual cortex _left	4	3.8686	-5.6795	5.3879	-6.1179
corpus callosum _right	4	3.5028	0.4067	5.5762	-0.8379
parietal cortex posterior area_right	4	4.0772	5.1437	5.6881	-5.6379
Thalamus_medial nucleus group_left	4	4.3296	-0.0042	4.6023	-9.9579
striatum_left	3	3.5007	-4.6729	8.5285	-1.3179
septal area _right	3	4.06	1.7322	9.3984	-0.3579
sensory cortex _left	3	3.7363	-5.6401	6.5092	1.0821
Thalamus_medial nucleus group_right	3	3.7586	0.5608	7.8317	-3.2379
capsule_right	3	3.7492	2.1367	7.1276	-0.8379
corpus callosum _left	3	3.7317	-4.3146	5.9595	1.5621
orbital cortex_left	3	3.9923	-3.5213	6.7637	3.0021
PFC_left	3	3.9923	-3.5213	6.7637	3.0021
supraoptic region_left	3	3.9319	-1.189	6.4357	-1.7979
Thalamus_lateral nucleus group_right	3	4.0387	1.64	6.7681	-3.7179
olfactory bulb_right	3	3.6674	1.2487	6.6461	-4.1979
anterior nucleus group _left	3	3.8565	-0.2548	6.2065	-1.7979
cerebellum _poaterior lobe of cerebellum_left	3	3.6717	-0.2482	6.3907	-2.2779
dentate gyrus_right	3	3.7647	0.0113	6.2641	-2.2779
supraoptic region_left	3	3.8258	-0.1098	6.2571	-2.2779
third ventricle_left	3	3.9314	-0.3736	4.9972	6.8421
hippocampus_right	3	3.7227	1.1212	6.3149	-13.7979

cerebellum _poaterior lobe of cerebellum_right	3	3.4867	0.2817	4.7877	-11.8779
bed nucleus of stria terminalis _left	2	3.7086	-4.6465	8.6989	-3.2379
septal area _left	2	3.6244	-4.3565	8.5169	-4.1979
septal area _left	2	3.589	-1.4815	8.845	0.6021
fourth ventricle _right	2	3.4505	3.8708	7.8691	0.1221
piriform cortex_right	2	3.3875	4.2621	8.5929	0.6021
amygdaloid body_right	2	3.4915	3.4952	8.5432	-2.7579
midbrain _ superior colliculus _left	2	3.7513	0.5674	8.1752	-3.7179
olfactory bulb_right	2	3.5134	0.4529	7.7683	-4.1979
sensory cortex _right	2	3.7389	4.2423	6.996	2.0421
Thalamus_medial nucleus group_left	2	3.449	-1.8373	7.1512	-3.7179
cerebellum _poaterior lobe of cerebellum_left	2	3.6194	-1.965	7.2432	-3.2379
cerebellum _poaterior lobe of cerebellum_left	2	4.008	-4.1828	6.0665	2.0421
midbrain_tegmentum of midbrain_left	2	3.8521	-3.4002	6.6114	3.0021
pfi flocculonodular lobe _left	2	3.7411	-4.3146	6.1011	1.5621
pontine _ basilar part of pons _left	2	3.4906	-4.5807	6.3444	2.0421
third ventricle_right	2	3.6181	0.0179	6.3067	-2.7579
olfactory bulb_right	2	3.4658	-0.2286	5.1894	6.3621
hippocampus_left	2	3.598	-5.6795	5.6888	-6.1179
hippocampus_left	2	4.8026	0.9828	6.3069	-13.7979
pontine _ tegmentum of pons_right	2	3.6074	1.6136	4.987	-1.7979
septal area _right	2	3.8266	1.752	5.4375	-1.7979
third ventricle_right	2	3.4626	1.607	5.3869	-1.3179
amygdaloid body_left	2	4.1529	-0.0108	5.1438	-9.4779
anterior commissure_right	2	4.2542	-0.0108	4.5597	-9.4779
pineal gland	2	3.4806	0.2355	5.0736	-8.5179
piriform cortex_left	1	3.3665	-4.8113	8.8214	-1.3179
medulla oblongata_right	1	3.7379	1.8772	9.3074	-0.8379
striatum_right	1	3.5191	1.8706	9.1232	-0.3579
pontine _ tegmentum of pons_right	1	3.6877	3.8642	8.1274	0.6021
cerebral peduncle _right	1	3.5887	3.3873	8.6214	-3.7179
cerebellum _anterior lobe of cerebellum _right	1	3.4387	-5.2488	6.9321	1.5621
corpus callosum _left	1	3.6421	-5.229	7.2015	0.1221
striatum_left	1	3.5926	-4.9522	7.5184	0.1221
supraoptic region_left	1	3.6021	-3.9586	7.9558	-4.1979
Thalamus_midline nucleus group_right	1	3.6511	0.5608	8.1326	-3.2379
cerebellum _cerebellar nucleus_right	1	3.6712	2.4003	7.2	0.1221
Thalamus_lateral nucleus group_left	1	3.3652	-0.7779	7.7121	-2.7579
Thalamus_medial nucleus group_right	1	3.425	-0.1098	7.4253	-2.2779
anterior nucleus group _right	1	3.3652	-0.1164	7.3827	-1.7979
striatum_left	1	3.367	-3.5081	6.548	2.0421
dentate gyrus_left	1	3.4221	-1.3274	6.1445	-1.7979
olfactory tract_right	1	3.3886	1.1103	6.6381	-4.1979
supraoptic region_right	1	3.3782	0.976	6.4043	-3.2379
cerebellum _anterior lobe of cerebellum _right	1	3.4465	0.0179	6.1474	-2.7579
dentate gyrus_left	1	3.4595	-0.3932	6.1985	-1.7979
third ventricle_left	1	3.6976	-0.2482	6.2491	-2.2779
Thalamus_medial nucleus group_left	1	3.4069	-0.2286	5.331	6.3621
auditory cortex_left	1	3.5277	-5.794	5.7244	-6.5979

retrosplenial cortex_right	1	3.7505	0.706	5.99	-13.7979
midbrain _ inferior colliculus _right	1	3.3701	0.62	4.8505	2.5221
retrosplenial cortex_left	1	3.4292	-0.8856	5.6148	-13.7979
pineal gland	1	3.4745	0.4684	4.1732	3.4821
midbrain_tegmentum of midbrain_right	1	3.7967	-0.0108	4.8429	-9.4779
motor cortex _right	1	3.5028	0.5189	4.9729	-8.9979

Table S3 The ALFF statistical analysis offers additional localization and quantitative details, including the volume of each brain region within the cluster, the t-value of the peak difference, and the coordinate position ($p < 0.01$, cluster size > 50). ALFF refers to the amplitude of low-frequency fluctuations.

Table S4 Brain regions showing abnormal FA in aged MCI rats

Brain regions	Ke	t value	MNI coordinates		
			X	Y	Z
**Postoperative day 2**					
**RUVBL2-shRNA-Sevo > Scramble-shRNA-Sevo**					
cerebellum _poaterior lobe of cerebellum_left	3547	11.5436	-3.0308	7.9688	-13.798
sensory cortex _right	3143	18.2085	2.9391	3.7116	-1.3179
cerebellum _anterior lobe of cerebellum _left	3058	12.3208	-3.6199	7.3138	-9.9579
striatum_left	3037	13.3096	-3.189	7.6914	-12.358
sensory cortex _left	3016	12.7743	-4.8334	6.0904	-8.5179
cerebellum _anterior lobe of cerebellum _right	2949	10.9727	-0.1692	6.0153	2.0421
pontine _ tegmentum of pons_left	2457	10.7956	-2.8375	8.2819	1.0821
cerebellum _poaterior lobe of cerebellum_right	2414	8.9227	1.6334	7.4512	-3.2379
striatum_right	2398	9.6067	1.7324	8.6743	-10.4379
Thalamus_lateral nucleus group_left	2081	12.6337	-2.5079	7.3289	-2.7579
pontine _ tegmentum of pons_right	2055	12.7427	0.6466	9.2705	-9.4779
Thalamus_lateral nucleus group_right	1864	9.6957	1.6268	7.5679	-2.7579
motor cortex _left	1674	10.5375	-4.7189	6.0548	-8.0379
hippocampus_left	1510	14.1458	-4.4487	6.4707	-7.5579
corpus callosum _left	1452	12.4921	-4.5871	6.1618	-7.5579
hippocampus_right	1400	10.1048	0.2945	5.2768	7.3221
corpus callosum _right	1395	12.3793	4.6404	6.1533	-8.0379
visual cortex _right	1392	10.212	4.9238	5.9287	-8.5179
motor cortex _right	1347	14.3119	4.7788	6.0197	-8.0379
PFC_right	1164	13.445	2.7654	7.8966	-7.5579
medulla oblongata_left	1093	10.7214	-1.9782	9.2112	-2.2779
PFC_left	1028	7.7492	-4.1958	6.5847	-7.0779
olfactory bulb_left	984	9.4509	-0.9165	5.3661	7.3221
midbrain_tegmentum of midbrain_right	958	14.5872	2.6336	8.0905	-8.0379
olfactory bulb_right	939	9.5126	0.4156	5.1422	7.3221
retrosplenial cortex_right	929	8.827	0.0309	4.4787	6.3621
cingulate gyrus _left	846	9.3232	-0.3076	5.5648	2.0421
midbrain_tegmentum of midbrain_left	818	10.6033	2.9038	7.763	-7.5579
visual cortex _left	810	9.2831	-3.5145	4.7724	-7.5579
retrosplenial cortex_left	804	9.3737	-0.3734	3.9899	-3.2379
cingulate gyrus _right	777	8.0588	-0.1626	5.6154	1.5621
septal area _right	750	11.3826	0.6532	9.4547	-9.9579
orbital cortex_right	721	13.445	2.7654	7.8966	-7.5579
auditory cortex_left	680	7.2373	-6.3567	5.2221	-4.6779
orbital cortex_left	663	7.7492	-4.1958	6.5847	-7.0779
accumbens nucleus_right	659	12.3585	1.1895	9.1848	-9.9579
septal area _left	641	10.7364	-4.7783	6.1139	-3.7179
medulla oblongata_right	638	11.0094	1.4884	7.4006	-2.7579
accumbens nucleus_left	635	9.9808	-2.6991	8.4315	1.0821
midbrain _ superior colliculus _right	629	6.885	0.2025	6.6129	-6.1179
midbrain _ inferior colliculus _right	621	7.0167	2.7588	7.4292	-7.0779
midbrain _ superior colliculus _left	602	6.696	-3.8177	6.6215	-5.6379
third ventricle_right	587	11.608	0.3011	5.3194	6.8421
olfactory cortex_left	581	16.2446	-4.7123	6.239	-8.5179

olfactory cortex_right	557	15.3465	5.0581	6.3041	-9.4779
third ventricle_left	551	13.6326	-4.8994	6.1069	-3.7179
cerebellum _cerebellar nucleus_left	521	11.3233	-3.1824	7.5924	-12.8379
pfi flocculonodular lobe _left	509	9.2774	-3.189	7.833	-12.3579
Thalamus_medial nucleus group_left	483	7.8775	-0.6461	7.0934	-2.2779
capsule_left	481	8.4065	-3.0374	7.7846	-13.3179
insular cortex_right	418	6.9289	5.357	8.3254	-2.2779
auditory cortex_right	412	9.7541	6.9923	7.4206	-4.1979
dentate gyrus_left	404	7.8337	-1.044	6.2031	-2.2779
pfi flocculonodular lobe _right	396	6.9781	4.9327	7.3886	-0.3579
cerebellum _cerebellar nucleus_right	377	7.4021	1.5938	6.3283	-0.3579
insular cortex_left	365	9.1681	-3.7847	6.9761	-8.0379
capsule_right	362	7.2525	2.8446	9.2928	-13.3179
midbrain _ inferior colliculus _left	352	7.2844	-2.4419	7.1708	-7.5579
amygdaloid body_right	337	7.2402	5.3702	8.4106	-3.2379
Thalamus_medial nucleus group_right	304	9.8917	-0.1692	6.1569	2.0421
dentate gyrus_right	296	6.561	1.9368	6.7719	-15.2379
prelimbic cortex_right	286	6.6821	-0.0547	6.1213	2.5221
piriform cortex_right	284	10.5807	2.7588	8.1549	-7.0779
hypothalamus_preoptic region_left	275	7.5905	-1.5735	9.8625	-12.8379
amygdaloid body_left	270	6.8258	-3.1826	9.0422	-2.7579
hypothalamus_tuberal region _left	247	7.8444	-1.5869	9.3332	-1.7979
bed nucleus of stria terminalis _left	241	9.8535	-4.6399	6.706	-3.7179
temporal association cortex_left	239	9.7925	-6.9852	5.7822	-8.0379
parietal cortex posterior area_left	236	9.2965	-5.9654	4.0343	-4.1979
piriform cortex_left	231	12.6134	-2.82	9.1695	-8.9979
frontal association cortex_left	221	6.5811	-1.4264	3.7532	5.4021
frontal association cortex_right	213	5.1343	0.1759	4.6709	5.8821
prelimbic cortex_left	207	6.8614	-0.4219	6.9295	-8.5179
ptpretectal area _left	206	6.0244	-2.077	7.264	-5.1579
supraoptic region_left	204	8.8301	-4.4949	6.7566	-4.1979
Thalamus_midline nucleus group_left	203	10.1696	-0.6393	8.7483	-12.8379
midbrain _ periaqueductal gray matter_left	198	7.4366	-0.8305	8.2579	-8.9979
fourth ventricle _left	194	6.5725	-0.4219	6.7879	-8.5179
midbrain _ periaqueductal gray matter_right	181	7.386	0.3275	5.7907	4.9221
parietal cortex posterior area_right	180	6.1194	5.6602	4.7063	-4.1979
temporal association cortex_right	171	9.088	6.1175	5.6968	-8.5179
hypothalamus_preoptic region_right	166	7.7001	0.2751	10.1436	-11.3979
Thalamus_midline nucleus group_right	143	5.602	0.1561	5.2688	7.3221
anterior nucleus group _left	143	7.6715	-1.169	8.4767	-13.3179
anterior commissure_left	139	7.4832	-2.0176	8.674	-9.4779
bed nucleus of stria terminalis _right	136	6.3604	1.2027	8.9868	-10.9179
tenia tecta_right	133	6.6519	1.8378	8.1861	-8.0379
parietal association cortex_right	129	6.2167	4.4426	4.5937	-3.7179
claustral layer _right	124	6.3543	4.2555	7.6653	1.0821
olfactory tubercle_left	124	11.6945	-2.6923	9.2191	-9.4779
ptpretectal area _right	120	6.6444	0.4222	5.3264	6.8421
fourth ventricle _right	110	5.0816	0.0971	7.6852	-8.5179
tenia tecta_left	107	9.653	-0.3008	8.6888	-8.5179

frontal cortex area 3_left	105	20.1412	-4.4487	6.3114	-7.5579
supraoptic region_right	102	5.9657	-0.123	8.5083	-1.3179
anterior commissure_right	101	9.7311	1.4465	8.7898	-8.5179
dorsal peduncular cortex_left	95	6.1884	-0.1624	7.8118	-8.5179
frontal cortex area 3_right	95	7.6868	4.9238	5.7694	-8.5179
hypothalamus_tuberal region _right	94	5.9543	2.1499	8.6819	-1.7979
olfactory tubercle_right	91	9.2989	0.6598	9.7805	-10.4379
dorsal peduncular cortex_right	88	5.8638	0.0971	8.1277	-8.5179
anterior nucleus group _right	87	6.9732	1.7454	6.5631	-1.3179
midbrain _ substantia nigra_left	80	9.0087	-1.9254	9.1095	-6.1179
pontine _ basilar part of pons _left	78	9.2331	-2.9584	9.0199	-8.9979
parietal association cortex_left	72	8.2237	-4.8994	3.7705	-3.7179
claustral layer _left	70	6.4103	-3.4881	7.704	-9.4779
infralimbic cortex_right	69	5.162	0.0837	6.4302	2.5221
pontine _ basilar part of pons _right	69	11.1192	1.3279	9.0512	-9.9579
cerebral peduncle _left	64	6.9255	-3.1628	8.5859	-4.1979
infralimbic cortex_left	63	6.8637	-0.8571	6.6168	3.0021
pineal gland	43	4.2287	-0.1822	5.206	-7.0779
olfactory tract_left	41	6.1443	-1.1034	6.9879	2.0421
olfactory tract_right	41	5.133	0.765	7.0959	2.0421
midbrain_tegmentum of midbrain_left	38	5.4202	-0.4417	9.2797	-7.0779
interstitial nucleus _right	33	5.5158	3.739	8.9303	-0.3579
interstitial nucleus _left	24	4.5672	-1.8528	8.8524	-11.3979
midbrain _ substantia nigra_right	24	4.0666	2.8708	8.7182	-5.1579
posterior commissure_right	24	6.2185	0.3211	6.7763	-4.6779
cerebral peduncle _right	23	4.8869	4.1831	7.4992	-3.7179
posterior commissure_left	21	5.4101	-0.6131	6.7223	-4.6779
retrosplenial cortex_left	21	4.2736	-1.0108	5.1079	-14.7579
sensory cortex _right	20	4.9979	5.6076	7.4469	-10.4379
motor cortex _left	18	4.4955	-1.8396	4.2648	-12.3579
midbrain _ substantia nigra_left	15	6.7743	-2.7517	8.3932	-5.1579
sensory cortex _left	14	4.8504	-2.3495	4.4042	-14.2779
piriform cortex_left	11	4.6271	-4.5279	9.1632	-1.7979
blood vessel _left	11	4.322	-2.998	6.4279	-6.1179
mammillary region_left	11	5.2053	-1.6857	9.2976	-4.6779
amygdaloid body_left	9	5.5407	-4.5345	9.1206	-1.3179
red nucleus_left	9	4.3365	-0.5933	8.9033	-6.1179
subthalamic nucleus_left	8	4.3008	-2.0968	8.8885	-3.7179
motor cortex _left	8	4.1687	-1.8132	4.7184	-14.2779
LC_left	7	6.4238	1.3013	6.3835	2.0421
pfi flocculonodular lobe _right	6	4.1568	5.4692	7.2973	-10.4379
corpus callosum _left	5	3.4735	-1.6789	5.3947	-15.2379
blood vessel _right	4	10.3164	-0.1692	5.8737	2.0421
Thalamus_medial nucleus group_left	4	3.5792	-0.202	9.1669	-5.6379
pontine _ tegmentum of pons_left	3	5.4625	-0.3206	9.2867	-7.0779
LC_right	2	3.629	-1.4747	7.1369	-9.9579
cerebellum _poaterior lobe of cerebellum_right	2	3.4479	5.4692	6.9964	-10.4379
cerebellum _poaterior lobe of cerebellum_left	2	3.3637	-1.0174	5.5078	-14.2779
motor cortex _left	2	3.3577	-1.5471	5.3601	-14.7579

sensory cortex _right	2	3.1886	3.2161	4.7558	-11.3979
subthalamic nucleus_right	1	3.5652	2.5676	8.5318	-3.2379
mammillary region_right	1	3.5846	-0.0702	9.4332	-5.1579
pontine _ tegmentum of pons_right	1	3.3645	-0.0504	9.2601	-6.5979
sensory cortex _left	1	3.1603	-2.2482	4.2834	-12.8379
visual cortex _left	1	3.1702	-3.4332	2.6106	5.4021

Table S4 The FA statistical analysis offers additional localization and quantitative details, including the volume of each brain region within the cluster, the t-value of the peak difference, and the coordinate position ($p < 0.01$, cluster size > 50). FA refers to the fractional anisotropy.

Table S5 Brain regions showing abnormal ALFF in aged MCI rats

Brain regions	Ke	t value	MNI coordinates		
			X	Y	Z
**Postoperative day 30**					
**RUVBL2-shRNA-Sevo > Scramble-shRNA-Sevo**					
retrosplenial cortex_right	259	6.2184	0.4486	3.4613	4.9221
cerebellum _poaterior lobe of cerebellum_right	209	5.551	1.2223	7.0598	-2.2779
cerebellum _anterior lobe of cerebellum _right	162	5.0236	0.2553	5.2014	-9.9579
motor cortex _right	150	5.9543	0.5936	4.2376	4.4421
visual cortex _left	150	5.5038	-1.8111	2.7888	4.4421
cingulate gyrus _right	135	4.7741	0.2553	5.343	-9.9579
midbrain _ superior colliculus _left	117	5.2927	-1.0153	4.5871	4.4421
Thalamus_medial nucleus group_right	104	5.2606	0.9562	6.8606	-1.7979
motor cortex _left	64	4.9589	-1.0153	4.7287	4.4421
cingulate gyrus _left	55	4.7349	-0.8703	4.4961	3.9621
anterior nucleus group _right	53	5.3115	1.2157	6.8756	-1.7979
PFC_left	52	4.4203	-1.4198	4.964	4.9221
Thalamus_medial nucleus group_left	50	5.3064	-0.3866	7.4093	-2.2779
frontal association cortex_right	48	5.4583	0.7188	4.4613	5.4021
cerebellum _poaterior lobe of cerebellum_left	45	5.9216	-0.5011	7.2856	-2.7579
pontine _ tegmentum of pons_left	43	6.6899	-0.6593	8.9021	-1.3179
visual cortex _right	42	4.9425	0.7254	2.8932	4.9221
cerebellum _anterior lobe of cerebellum _left	40	4.029	-0.446	3.9461	2.0421
visual cortex _right	39	4.1436	3.0247	3.8213	2.5221
retrosplenial cortex_left	38	4.5769	-1.4264	3.0098	5.4021
midbrain _ superior colliculus _right	36	5.8835	0.7254	4.3446	4.9221
supraoptic region_right	34	6.2675	0.6728	6.802	-1.3179
retrosplenial cortex_left	34	4.3451	-1.0285	4.201	5.4021
cingulate gyrus _right	33	4.6497	0.1299	5.2593	-0.8379
motor cortex _right	32	4.2459	1.3543	5.8586	-11.8779
third ventricle_right	31	5.8106	0.6662	6.7594	-0.8379
orbital cortex_left	30	4.0351	-1.4264	4.9214	5.4021
supraoptic region_left	29	5.3641	-0.7911	8.7951	-1.7979
striatum_left	29	3.8957	-2.7123	5.7267	2.0421
frontal association cortex_left	27	4.438	-1.0285	4.3603	5.4021
olfactory bulb_left	27	3.9474	-0.7649	5.1584	6.3621
cerebellum _anterior lobe of cerebellum _left	24	3.7787	-2.7123	5.8683	2.0421
pineal gland	24	4.7622	0.2023	3.8147	3.9621
septal area _left	23	3.7261	-1.0704	6.3159	-0.3579
prelimbic cortex_left	22	4.4203	-1.4198	4.964	4.9221
Thalamus_lateral nucleus group_right	21	5.216	1.3607	7.2094	-2.2779
capsule_right	20	4.2726	2.4401	8.059	-12.8379
Thalamus_midline nucleus group_right	20	3.8593	0.9892	6.4895	-4.1979
motor cortex _left	20	4.8099	-4.6071	4.2801	3.9621
capsule_right	19	4.8445	2.6796	7.5021	-1.3179
Thalamus_lateral nucleus group_right	19	4.2006	2.4467	8.1016	-13.3179
cerebellum _poaterior lobe of cerebellum_right	18	4.4831	2.6796	7.6437	-1.3179
cerebellum _poaterior lobe of cerebellum_right	18	4.3447	2.4467	8.2432	-13.3179
pfi flocculonodular lobe _right	18	3.8582	2.5785	7.9254	-12.8379

pontine _ tegmentum of pons_left	18	4.063	-1.367	7.1987	1.0821
septal area _right	18	3.9188	0.6596	6.7168	-0.3579
cerebellum _poaterior lobe of cerebellum_right	18	4.2563	-0.0085	5.3929	-0.8379
motor cortex _left	18	3.8076	-1.5079	4.0018	2.5221
Thalamus_lateral nucleus group_right	17	4.7107	2.5478	7.5367	-1.7979
cerebellum _anterior lobe of cerebellum _right	17	4.8546	0.8112	6.6507	-1.3179
motor cortex _right	17	3.9872	2.8797	3.9123	3.0021
insular cortex_right	16	4.2615	5.6074	8.171	-0.3579
striatum_right	16	4.1517	2.673	7.4595	-0.8379
bed nucleus of stria terminalis _right	16	4.0118	1.0641	7.0656	-0.8379
cerebellum _anterior lobe of cerebellum _left	16	3.8084	-0.9493	6.1813	-0.3579
third ventricle_right	16	4.5703	2.0356	6.8252	-12.3579
cerebellum _anterior lobe of cerebellum _left	16	3.6774	-1.5079	3.8425	2.5221
striatum_right	15	3.7918	2.1169	8.168	0.6021
cerebellum _anterior lobe of cerebellum _right	15	4.3204	0.2287	3.401	2.0421
olfactory bulb_right	15	3.4291	1.2419	4.6903	6.3621
cerebellum _anterior lobe of cerebellum _left	15	3.8202	-0.1362	5.3433	-0.3579
hypothalamus_preoptic region_left	14	3.9485	-0.5275	9.0268	-0.8379
cerebellum _poaterior lobe of cerebellum_right	14	4.5329	1.3675	7.5545	-12.8379
cerebellum _anterior lobe of cerebellum _right	14	3.998	0.1299	5.1177	-0.8379
cerebellum _poaterior lobe of cerebellum_left	14	3.6983	-0.123	5.4285	-1.3179
septal area _right	13	4.0248	1.3675	7.4129	-12.8379
blood vessel _left	13	3.5755	0.0705	3.2652	3.4821
cerebellum _anterior lobe of cerebellum _right	13	4.1523	1.8906	6.9162	-11.8779
striatum_right	13	4.0196	2.029	7.0658	-11.8779
hippocampus_left	12	3.7666	-4.2222	8.6091	-5.1579
hippocampus_right	12	3.6832	1.2419	4.8319	6.3621
visual cortex _left	12	4.5597	-4.6005	4.4643	3.4821
pfi flocculonodular lobe _right	11	4.0373	2.5478	7.3774	-1.7979
pontine _ tegmentum of pons_right	11	4.09	2.5478	7.6783	-1.7979
cerebellum _anterior lobe of cerebellum _right	11	4.2145	1.3609	7.5119	-12.3579
striatum_right	10	3.6922	2.693	7.7305	-12.3579
striatum_left	10	4.0623	-1.4881	7.0501	1.0821
third ventricle_right	10	3.9712	1.3675	7.6961	-12.8379
insular cortex_left	10	4.3629	-1.0219	4.2436	4.9221
mammillary region_left	9	3.8519	-0.6131	9.3596	-4.6779
hypothalamus_tuberal region _left	9	4.8677	-0.6593	8.7605	-1.3179
striatum_right	9	3.6962	3.5193	8.7477	-13.3179
septal area _left	9	3.9849	-1.3604	7.0997	0.6021
anterior nucleus group _left	9	4.2585	-0.3932	7.3667	-1.7979
cerebellum _poaterior lobe of cerebellum_left	9	3.4671	-0.8109	6.1893	-0.3579
cerebellum _poaterior lobe of cerebellum_right	9	4.1776	1.3543	6.0179	-11.8779
fourth ventricle _right	8	4.2879	2.673	7.6011	-0.8379
medulla oblongata_right	8	4.1629	2.6862	7.6863	-1.7979
medulla oblongata_right	8	4.0758	2.3083	8.5361	-13.3179
medulla oblongata_right	8	4.3291	1.4818	7.358	-2.2779
cingulate gyrus _right	8	4.0753	0.4618	4.2722	3.9621
insular cortex_right	8	5.4789	0.587	4.3366	4.9221
corpus callosum _left	8	4.8146	-4.6071	4.4217	3.9621

visual cortex _left	8	3.6296	-3.9629	2.9054	4.9221
pontine _ tegmentum of pons_right	7	3.8758	2.1235	8.069	0.1221
midbrain _ inferior colliculus _left	7	4.0527	-0.8637	4.3794	3.4821
cingulate gyrus _left	7	3.4412	-0.2878	5.4094	0.6021
dentate gyrus_left	6	3.7411	-4.3433	8.6021	-5.1579
medulla oblongata_right	6	3.6876	2.1169	8.0264	0.6021
midbrain_tegmentum of midbrain_right	6	4.1795	0.6992	6.6715	-3.2379
pontine _ tegmentum of pons_right	6	3.383	1.0641	7.5081	-0.8379
anterior nucleus group _right	6	3.6675	1.3741	7.4555	-13.3179
cerebellum _cerebellar nucleus_right	6	4.4301	1.8972	6.9588	-12.3579
cerebellum _poaterior lobe of cerebellum_right	6	4.8733	2.0356	6.9668	-12.3579
fourth ventricle _left	5	3.5976	-1.4815	6.9334	0.6021
Thalamus_midline nucleus group_left	5	3.4854	-0.3734	7.1936	-3.2379
medulla oblongata_left	5	3.5956	-0.3866	7.5509	-2.2779
Thalamus_medial nucleus group_left	5	3.8892	-0.1296	5.3859	-0.8379
hippocampus_right	5	3.3111	-0.1032	5.9988	-2.7579
medulla oblongata_left	4	3.636	-0.7845	8.6961	-2.2779
midbrain_tegmentum of midbrain_left	4	3.4004	-0.4064	8.8745	-0.8379
pfi flocculonodular lobe _right	4	3.6948	3.5127	8.8467	-12.8379
amygdaloid body_left	4	3.4154	-0.2548	7.0738	-1.7979
olfactory bulb_right	4	4.0302	0.9892	6.6311	-4.1979
corpus callosum _right	4	3.4615	1.6596	3.8145	4.9221
dentate gyrus_right	4	3.3067	-0.0966	5.7582	-3.2379
capsule_right	3	3.5338	3.3809	8.7397	-13.3179
cerebellum _cerebellar nucleus_left	3	3.4391	-1.4881	6.8908	1.0821
anterior nucleus group _left	3	3.3156	-1.334	6.3851	-1.3179
cerebellum _poaterior lobe of cerebellum_left	3	3.1658	-3.1036	5.587	1.5621
third ventricle_left	3	3.3745	-0.5054	5.315	6.3621
hippocampus_left	3	3.2232	-0.235	5.7502	-3.2379
midbrain _ inferior colliculus _left	3	3.5676	-1.5145	4.1008	3.0021
accumbens nucleus_right	2	3.3874	2.2487	8.1334	1.0821
cerebellum _poaterior lobe of cerebellum_right	2	3.7245	3.3809	8.8813	-13.3179
cerebellum _anterior lobe of cerebellum _right	2	3.3756	2.5785	8.2086	-12.8379
fourth ventricle _right	2	4.0565	2.3017	8.4935	-12.8379
cerebellum _anterior lobe of cerebellum _right	2	3.6118	-2.7057	5.9109	1.5621
corpus callosum _left	2	3.6309	-2.8573	5.6761	2.5221
midbrain _ inferior colliculus _left	2	3.3283	-2.8573	5.5168	2.5221
hippocampus_left	2	3.435	-0.7715	4.9742	6.8421
midbrain _ inferior colliculus _right	2	4.2844	0.7386	4.4298	3.9621
corpus callosum _right	2	3.1564	-0.0085	5.5522	-0.8379
dentate gyrus_left	2	3.1898	-0.2416	5.7076	-2.7579
sensory cortex _left	2	3.3357	-4.5939	4.3653	3.0021
Thalamus_medial nucleus group_left	2	3.3931	-0.0042	5.1864	-9.9579
corpus callosum _right	2	3.6089	1.3609	5.9012	-12.3579
midbrain_tegmentum of midbrain_left	1	3.3114	-0.7449	9.3942	-5.1579
bed nucleus of stria terminalis _left	1	3.2011	-0.9361	8.4613	-1.3179
septal area _left	1	3.2218	-4.3565	8.5169	-4.1979
interstitial nucleus _right	1	3.4121	1.9851	8.2026	0.1221
pfi flocculonodular lobe _right	1	3.3571	5.6008	7.9691	0.1221

cerebellum _cerebellar nucleus_right	1	3.3313	2.693	7.5889	-12.3579
pontine _ tegmentum of pons_right	1	3.5688	2.4467	8.5441	-13.3179
accumbens nucleus_left	1	3.4351	-1.367	7.3403	1.0821
dentate gyrus_right	1	3.2587	1.2223	6.3341	-2.2779
fourth ventricle _right	1	3.2284	1.1893	7.13	0.1221
hippocampus_right	1	3.1813	1.4818	6.0482	-2.2779
olfactory tract_right	1	3.8047	1.1103	6.6381	-4.1979
pfi flocculonodular lobe _right	1	3.1521	1.4818	5.9066	-2.2779
third ventricle_left	1	3.3575	-1.3406	6.6434	-0.8379
amygdaloid body_right	1	3.1652	-0.1164	5.4711	-1.7979
retrosplenial cortex_right	1	3.1561	0.1431	5.3445	-1.7979
hippocampus_left	1	3.5878	-4.4621	4.4723	3.4821
retrosplenial cortex_left	1	3.2135	-4.5939	4.5069	3.0021

Table S5 The ALFF statistical analysis offers additional localization and quantitative details, including the volume of each brain region within the cluster, the t-value of the peak difference, and the coordinate position ($p < 0.01$, cluster size > 50). ALFF refers to the amplitude of low-frequency fluctuations.

Table S6 Brain regions showing abnormal FA in aged MCI rats

Brain regions	Ke	t value	MNI coordinates		
			X	Y	Z
**Postoperative day 30**					
**RUVBL2-shRNA-Sevo > Scramble-shRNA-Sevo**					
Thalamus_lateral nucleus group_left	81	4.7309	-3.9454	6.2887	-5.1579
pontine _ tegmentum of pons_left	68	5.5833	-1.1954	9.032	-11.3979
auditory cortex_right	61	4.8489	7.1241	7.0851	-3.7179
medulla oblongata_left	44	4.821	-1.7185	9.0862	-12.3579
sensory cortex _right	39	4.0966	6.9593	6.3226	-1.7979
bed nucleus of stria terminalis _left	29	6.0931	-1.1954	9.1736	-11.3979
accumbens nucleus_left	26	6.0353	-1.202	9.131	-10.9179
olfactory cortex_left	24	4.9841	-6.8732	8.2394	-6.1179
midbrain_tegmentum of midbrain_left	18	7.9337	-0.7187	7.0673	3.0021
cerebellum _anterior lobe of cerebellum _right	17	4.2055	1.3013	6.3835	2.0421
olfactory bulb_right	16	4.0094	0.0509	6.3604	-5.1579
olfactory cortex_left	16	4.9848	-6.1894	6.1114	-8.0379
anterior commissure_left	15	4.2212	-1.7251	8.902	-11.8779
cerebellum _poaterior lobe of cerebellum_left	14	4.4727	-1.169	8.3351	-13.3179
hypothalamus_preoptic region_left	14	5.7515	-1.7185	9.2278	-12.3579
striatum_left	13	5.3282	-1.057	9.1816	-11.3979
dorsal peduncular cortex_left	13	5.5051	-0.8637	6.8751	3.4821
PFC_left	13	5.5051	-0.8637	6.8751	3.4821
sensory cortex _right	13	4.7436	4.2755	7.3699	-10.4379
striatum_right	13	4.8444	1.3013	6.2419	2.0421
pontine _ tegmentum of pons_right	12	5.1753	0.7784	8.935	-8.9979
cerebellum _poaterior lobe of cerebellum_right	12	4.7685	4.4139	7.2363	-10.4379
pontine _ tegmentum of pons_left	11	4.9045	-2.5607	8.882	1.0821
supraoptic region_left	10	4.6931	-1.4351	8.4191	-12.8379
accumbens nucleus_right	10	4.0507	0.9168	8.8014	-8.9979
hippocampus_left	9	4.5241	-5.308	5.8246	-4.1979
amygdaloid body_left	8	3.7671	-5.6993	9.3488	-4.6779
piriform cortex_left	8	3.8852	-6.7546	8.4205	-4.6779
striatum_left	8	5.232	-2.4396	8.7474	1.0821
septal area _left	8	3.8305	-4.5015	6.1299	-3.7179
anterior nucleus group _left	7	5.06	-1.4285	8.4617	-13.3179
hypothalamus_preoptic region_left	7	5.0422	-0.4196	7.7804	0.1221
posterior commissure_right	7	3.9435	0.1827	6.7683	-4.6779
insular cortex_right	6	3.684	6.4362	8.1291	-2.7579
hippocampus_right	6	3.6375	3.5084	5.1238	-3.7179
olfactory tubercle_right	5	3.9517	0.7784	9.0766	-8.9979
midbrain_tegmentum of midbrain_left	5	5.4362	-0.2812	7.93	0.1221
olfactory cortex_right	5	3.684	6.4494	8.2143	-3.7179
tenia tecta_left	5	5.7043	-0.7187	7.2089	3.0021
accumbens nucleus_left	4	5.5828	-2.5607	8.7404	1.0821
pontine _ tegmentum of pons_left	4	3.7959	-0.413	7.9646	-0.3579
septal area _left	4	4.3217	-0.4262	7.7378	0.6021
corpus callosum _right	4	5.5667	4.4205	7.4205	-10.9179
pfi flocculonodular lobe _right	4	4.9221	4.2821	7.5541	-10.9179

striatum_right	4	4.6118	4.2821	7.4125	-10.9179
midbrain _ superior colliculus _right	4	4.3079	0.0575	6.5623	-5.6379
corpus callosum _right	4	3.6778	3.5018	5.0812	-3.2379
midbrain_tegmentum of midbrain_right	3	3.7468	0.6334	8.8844	-8.5179
anterior commissure_left	3	4.1941	-0.5407	7.7734	0.1221
hypothalamus_preoptic region_right	3	4.1172	-0.1428	7.7964	0.1221
septal area _right	3	4.668	-0.1428	7.938	0.1221
midbrain _ periaqueductal gray matter_left	3	5.7675	-0.8637	7.0167	3.4821
hippocampus_left	3	3.7413	-4.3565	6.1805	-4.1979
LC_left	3	4.2055	1.3013	6.3835	2.0421
parietal association cortex_right	3	3.4906	3.6468	4.8486	-3.7179
capsule_left	2	3.8191	-1.8462	8.895	-11.8779
interstitial nucleus _left	2	3.751	-1.8528	8.8524	-11.3979
olfactory bulb_right	2	4.166	0.9102	8.7588	-8.5179
pontine _ basilar part of pons _right	2	3.4342	1.1895	9.0432	-9.9579
fourth ventricle _left	2	3.4613	-0.4262	7.5785	0.6021
midbrain_tegmentum of midbrain_right	2	3.8444	-0.1362	7.9806	-0.3579
sensory cortex _right	2	3.4857	6.8539	5.9435	-4.1979
cerebellum _anterior lobe of cerebellum _right	2	3.9057	4.2755	7.5115	-10.4379
Thalamus_medial nucleus group_left	2	4.1043	-0.0636	6.5553	-5.6379
corpus callosum _left	2	3.5602	-5.4225	5.2761	-4.6779
temporal association cortex_left	2	3.9936	-6.5873	5.8052	-8.0379
cerebellum _cerebellar nucleus_left	1	3.3853	-3.5871	6.7818	-2.2779
cerebellum _poaterior lobe of cerebellum_left	1	3.5862	-2.9124	7.104	-2.2779
corpus callosum _right	1	3.5626	1.2947	6.1993	2.5221
Thalamus_midline nucleus group_right	1	3.3714	0.4595	7.0852	-4.6779
anterior commissure_right	1	3.4518	-0.0702	6.5127	-5.1579
hypothalamus_tuberal region _left	1	3.4097	-0.0834	6.7107	-4.1979
orbital cortex_right	1	3.7694	0.0641	6.4456	-6.1179
third ventricle_right	1	3.5447	0.0377	6.7177	-4.1979
PFC_right	1	3.7694	0.0641	6.4456	-6.1179
sensory cortex _right	1	3.4171	3.6402	4.806	-3.2379

Table S6 The FA statistical analysis offers additional localization and quantitative details, including the volume of each brain region within the cluster, the t-value of the peak difference, and the coordinate position ($p < 0.01$, cluster size > 50). FA refers to the fractional anisotropy.

Table S7 Brain regions showing abnormal ALFF in aged MCI rats

Brain regions	Ke	t value	MNI coordinates		
			X	Y	Z
**Scramble-shRNA-Sevo Group**					
**Postoperative day 2 vs. Postoperative day 30**					
amygdaloid body_left	109	5.1957	-3.4553	8.942	-1.7979
midbrain _ periaqueductal gray matter_left	74	6.1578	-0.7185	6.9273	-7.0779
medulla oblongata_left	58	5.1167	-3.7321	9.0676	-1.7979
pontine _ tegmentum of pons_right	55	5.1768	0.9364	8.3258	-0.3579
PFC_left	55	6.2741	-0.7251	7.0263	-6.5979
orbital cortex_left	52	6.2741	-0.7251	7.0263	-6.5979
midbrain_tegmentum of midbrain_right	42	4.8187	1.0024	8.6102	-5.1579
pontine _ tegmentum of pons_right	38	4.5669	0.2553	10.4583	-9.9579
pontine _ tegmentum of pons_left	38	4.5686	-1.3495	9.697	-8.9979
olfactory bulb_left	32	4.3993	-0.1954	7.3156	-6.1179
olfactory tubercle_left	29	4.634	-1.3429	9.8812	-9.4779
midbrain_tegmentum of midbrain_left	27	3.9905	-1.011	8.7525	-4.6779
pontine _ tegmentum of pons_left	26	5.1014	-3.5937	9.0756	-1.7979
medulla oblongata_right	26	3.9754	2.561	9.2149	-2.7579
hypothalamus_preoptic region_right	26	4.9507	0.9364	8.4851	-0.3579
mammillary region_left	22	3.7449	-0.7515	9.0507	-4.6779
midbrain _ substantia nigra_right	21	4.8335	0.5872	8.8871	-5.1579
pontine _ tegmentum of pons_left	19	4.1269	-0.281	10.4273	-9.9579
red nucleus_right	18	4.4013	0.864	8.6022	-5.1579
midbrain_tegmentum of midbrain_left	17	3.7622	-0.4417	7.8283	-7.0779
supraoptic region_right	15	3.9035	2.561	9.3742	-2.7579
striatum_left	15	4.4437	-4.1696	7.1783	1.0821
midbrain _ periaqueductal gray matter_right	15	4.5021	-0.0702	7.0968	-5.1579
midbrain_tegmentum of midbrain_right	13	5.6781	0.2487	10.4157	-9.4779
midbrain_tegmentum of midbrain_left	13	4.4989	-1.3495	9.9802	-8.9979
bed nucleus of stria terminalis _right	13	4.2187	0.9364	8.1842	-0.3579
supraoptic region_left	12	4.259	-2.9124	9.0156	-2.2779
olfactory bulb_right	12	5.3081	0.0575	7.288	-5.6379
hypothalamus_tuberal region _left	11	3.9067	-3.1892	8.6987	-2.2779
accumbens nucleus_right	10	3.6666	0.7848	7.8078	0.6021
fourth ventricle _left	9	4.0311	-4.3014	7.0713	0.6021
midbrain_tegmentum of midbrain_left	8	4.6402	-0.1492	10.3927	-9.4779
mammillary region_right	8	3.9907	0.3211	8.8295	-4.6779
Thalamus_medial nucleus group_left	7	5.1352	-0.0636	7.1394	-5.6379
hypothalamus_tuberal region _right	6	3.6563	2.6928	9.0387	-2.2779
midbrain _ substantia nigra_left	5	3.5969	-0.7449	9.0933	-5.1579
pontine _ tegmentum of pons_left	5	3.8384	-4.0312	7.3279	1.0821
Thalamus_medial nucleus group_left	4	3.8298	-0.0042	10.4433	-9.9579
pontine _ basilar part of pons _right	4	4.7708	-0.0174	10.3581	-8.9979
capsule_right	4	3.8133	2.561	9.0733	-2.7579
anterior commissure_right	4	3.2956	0.798	7.893	-0.3579
striatum_left	3	3.4266	-0.8239	9.3271	-9.4779
medulla oblongata_left	3	3.3056	-0.7647	8.8239	-3.7179
striatum_right	3	3.3519	0.7848	8.3919	0.6021

pfi flocculonodular lobe _left	3	3.7813	-4.1696	7.0367	1.0821
prelimbic cortex_left	3	4.1071	-0.7119	6.8283	-7.5579
accumbens nucleus_left	2	3.268	-1.3363	9.6229	-9.9579
capsule_left	2	3.6576	-2.7847	8.7643	-2.7579
red nucleus_left	2	3.364	-1.1428	8.4862	-5.1579
septal area _right	2	3.6144	0.6464	7.7998	0.6021
cerebellum _anterior lobe of cerebellum _left	2	3.2785	-4.0312	7.0447	1.0821
supraoptic region_left	2	3.8382	-0.0504	7.2246	-6.5979
septal area _left	1	3.2263	-0.8173	9.3697	-9.9579
pontine _ basilar part of pons _left	1	3.2699	-3.209	9.2966	-0.8379
pontine _ tegmentum of pons_right	1	3.2474	2.6928	9.4812	-2.2779
hypothalamus_tuberal region _left	1	3.5288	-1.0176	8.7099	-4.1979
supraoptic region_left	1	3.3152	-0.0768	8.8065	-4.6779
medulla oblongata_right	1	3.3082	0.943	8.0852	-0.8379
claustral layer _left	1	3.3531	-4.0378	7.1437	1.5621
Thalamus_medial nucleus group_right	1	3.2573	-0.0768	7.3374	-4.6779
olfactory tract_left	1	3.1594	-0.7251	7.9113	-6.5979

Table S7 The ALFF statistical analysis offers additional localization and quantitative details, including the volume of each brain region within the cluster, the t-value of the peak difference, and the coordinate position ($p < 0.01$, cluster size > 50). ALFF refers to the amplitude of low-frequency fluctuations.

Table S8 Brain regions showing abnormal FA in aged MCI rats

Brain regions	Ke	t value	MNI coordinates		
			X	Y	Z
**Scramble-shRNA-Sevo Group**					
**Postoperative day 2 vs. Postoperative day 30**					
olfactory cortex_right	14	8.2597	6.1109	7.7074	-8.0379
dentate gyrus_right	7	4.3947	3.9632	8.6248	-6.5979
hippocampus_right	2	3.6966	4.6272	8.4045	-7.0779

Table S8 The FA statistical analysis offers additional localization and quantitative details, including the volume of each brain region within the cluster, the t-value of the peak difference, and the coordinate position ($p < 0.01$, cluster size > 50). FA refers to the fractional anisotropy.

Table S9 Brain regions showing abnormal ALFF in aged MCI rats

Brain regions	Ke	t value	MNI coordinates		
			X	Y	Z
**RUVBL2-shRNA-Sevo Group**					
**Postoperative day 2 vs. Postoperative day 30**					
mammillary region_right	18	4.1031	0.3279	9.5994	-15.2379
midbrain _ superior colliculus _right	17	4.1793	0.3343	5.9942	-5.6379
hypothalamus_tuberal region _right	13	3.9963	1.141	10.2305	-15.2379
frontal association cortex_right	12	4.8694	0.1959	5.8269	-5.6379
orbital cortex_right	10	4.4195	0.2025	5.8695	-6.1179
PFC_right	10	4.4195	0.2025	5.8695	-6.1179
medulla oblongata_right	7	3.6075	1.1476	10.1315	-15.7179
medulla oblongata_right	7	3.9776	0.3345	9.5004	-15.7179
insular cortex_right	7	4.5397	0.0641	5.7199	-6.1179
pontine _ tegmentum of pons_right	6	3.8538	1.0026	10.0809	-15.2379
olfactory bulb_right	5	4.0916	0.5938	6.1508	-5.6379
pontine _ tegmentum of pons_right	4	4.2351	0.3279	9.4578	-15.2379
retrosplenial cortex_right	3	4.4483	0.1959	5.6853	-5.6379
hippocampus_right	2	3.3625	0.3277	5.9516	-5.1579
Thalamus_midline nucleus group_right	1	3.1763	0.3213	9.2736	-14.7579
midbrain_tegmentum of midbrain_right	1	3.2768	0.6047	9.6154	-15.2379
ptpretectal area _right	1	3.6066	0.5872	6.2498	-5.1579

Table S9 The ALFF statistical analysis offers additional localization and quantitative details, including the volume of each brain region within the cluster, the t-value of the peak difference, and the coordinate position ($p < 0.01$, cluster size > 50). ALFF refers to the amplitude of low-frequency fluctuations.

Table S10 Brain regions showing abnormal FA in aged MCI rats

Brain regions	Ke	t value	MNI coordinates		
			X	Y	Z
**RUVBL2-shRNA-Sevo Group**					
**Postoperative day 2 vs. Postoperative day 30**					
midbrain_tegmentum of midbrain_left	15	4.2698	-1.4945	9.9296	-8.5179
pontine _ basilar part of pons _left	5	3.8298	-1.5011	9.887	-8.0379
pontine _ tegmentum of pons_left	9	3.8489	-1.235	9.9446	-8.5179

Table S10 The FA statistical analysis offers additional localization and quantitative details, including the volume of each brain region within the cluster, the t-value of the peak difference, and the coordinate position ($p < 0.01$, cluster size > 50). FA refers to the fractional anisotropy.
